# Burning questions for a warming and changing world: 15 unknowns in plant abiotic stress

**DOI:** 10.1093/plcell/koac263

**Published:** 2022-08-26

**Authors:** Paul E Verslues, Julia Bailey-Serres, Craig Brodersen, Thomas N Buckley, Lucio Conti, Alexander Christmann, José R Dinneny, Erwin Grill, Scott Hayes, Robert W Heckman, Po-Kai Hsu, Thomas E Juenger, Paloma Mas, Teun Munnik, Hilde Nelissen, Lawren Sack, Julian I Schroeder, Christa Testerink, Stephen D Tyerman, Taishi Umezawa, Philip A Wigge

**Affiliations:** Institute of Plant and Microbial Biology, Academia Sinica, Taipei 11529, Taiwan; Department of Botany and Plant Sciences, Center for Plant Cell Biology, University of California, Riverside, California 92521, USA; School of the Environment, Yale University, New Haven, Connecticut 06511, USA; Department of Plant Sciences, University of California, Davis, California 95616, USA; Department of Biosciences, University of Milan, Milan 20133, Italy; School of Life Sciences, Technical University Munich, Freising-Weihenstephan 85354, Germany; Department of Biology, Stanford University, Stanford, California 94305, USA; School of Life Sciences, Technical University Munich, Freising-Weihenstephan 85354, Germany; Laboratory of Plant Physiology, Plant Sciences Group, Wageningen University and Research, Wageningen 6708 PB, The Netherlands; Department of Integrative Biology, University of Texas at Austin, Austin, Texas 78712, USA; Department of Cell and Developmental Biology, School of Biological Sciences, University of California San Diego, La Jolla, California 92093, USA; Department of Integrative Biology, University of Texas at Austin, Austin, Texas 78712, USA; Centre for Research in Agricultural Genomics (CRAG), CSIC-IRTA-UAB-UB, Barcelona 08193, Spain; Consejo Superior de Investigaciones Científicas (CSIC), Barcelona 08028, Spain; Department of Plant Cell Biology, Green Life Sciences Cluster, Swammerdam Institute for Life Sciences, University of Amsterdam, Amsterdam NL-1098XH, The Netherlands; Department of Plant Biotechnology and Bioinformatics, Ghent University, Ghent 9052, Belgium; VIB Center for Plant Systems Biology, Ghent 9052, Belgium; Department of Ecology and Evolutionary Biology, Institute of the Environment and Sustainability, University of California, Los Angeles, California 90095, USA; Department of Cell and Developmental Biology, School of Biological Sciences, University of California San Diego, La Jolla, California 92093, USA; Laboratory of Plant Physiology, Plant Sciences Group, Wageningen University and Research, Wageningen 6708 PB, The Netherlands; ARC Center Excellence, Plant Energy Biology, School of Agriculture Food and Wine, University of Adelaide, Adelaide, South Australia 5064, Australia; Faculty of Agriculture, Tokyo University of Agriculture and Technology, Tokyo 6708 PB, Japan; Leibniz-Institut für Gemüse- und Zierpflanzenbau, Großbeeren 14979, Germany; Institute of Biochemistry and Biology, University of Potsdam, Potsdam 14476, Germany

## Abstract

We present unresolved questions in plant abiotic stress biology as posed by 15 research groups with expertise spanning eco-physiology to cell and molecular biology. Common themes of these questions include the need to better understand how plants detect water availability, temperature, salinity, and rising carbon dioxide (CO_2_) levels; how environmental signals interface with endogenous signaling and development (e.g. circadian clock and flowering time); and how this integrated signaling controls downstream responses (e.g. stomatal regulation, proline metabolism, and growth versus defense balance). The plasma membrane comes up frequently as a site of key signaling and transport events (e.g. mechanosensing and lipid-derived signaling, aquaporins). Adaptation to water extremes and rising CO_2_ affects hydraulic architecture and transpiration, as well as root and shoot growth and morphology, in ways not fully understood. Environmental adaptation involves tradeoffs that limit ecological distribution and crop resilience in the face of changing and increasingly unpredictable environments. Exploration of plant diversity within and among species can help us know which of these tradeoffs represent fundamental limits and which ones can be circumvented by bringing new trait combinations together. Better defining what constitutes beneficial stress resistance in different contexts and making connections between genes and phenotypes, and between laboratory and field observations, are overarching challenges.

## Introduction

### (By Paul E. Verslues, editor)

“Before now, you just needed to know the answers to the questions you were given; now you need to know questions which you have not been given and for which there is no answer.” This is the advice that many mentors have given, in one form or another, to those new to research. Here, we put ourselves to this test and present what several groups of scientists working in plant abiotic stress biology consider to be big questions for future research. This is a timely topic as climate change will bring about not just a general warming but also instability and extreme weather events of many types. Thus, climate change can increase the frequency and severity of single, combined, and even sequential abiotic stresses including drought, salinity, flooding, and even freezing. Rising carbon dioxide (CO_2_) levels can also directly influence how plants respond to these stresses. A common theme of plant stress research is that we are trying to understand how plants respond to excesses: too hot, too cold, too little water, too much water, too much light, too little light, or too much salt. These excesses are gradually (or not so gradually in some cases) becoming more of the norm for plants in many parts of the world. In this article, we focus on questions of fundamental plant biology and how stress affects physiological and molecular processes and adaptation, rather than climate change mitigation strategies discussed by our colleagues. Ultimately, we want to use our physiological and molecular knowledge to both predict the effect of climate change on plants and intervene to improve those outcomes, particularly in terms of ecosystem resiliency or crop yield. Thus, perhaps one overriding challenge is the question of scale and how to move our knowledge from one scale to another. How does the opening and closing of a membrane channel that occurs at a time scale of seconds (or less) influence growth responses that occur over days? How do those growth responses affect yield or reproductive fitness which is the culmination of months, or more, of the plant life cycle? How does knowing where a gene is expressed at the cellular scale, or where a protein is localized at the subcellular scale, help us understand coordination of root and shoot responses at the whole plant scale?

Another common theme that emerges from our big questions is the challenge for measurements of stress phenotypes to keep pace with, and make best use of, the ever-increasing amount of genomic data. How can this growing body of genomics data help us to understand gene and protein function and, ultimately, deploy that knowledge for plant improvement or understanding natural systems? Despite advances such as automated plant phenotyping and image analysis systems, this “phenotype gap” (Miflin, 2000) continues to grow larger as -omics data accumulate. In the process of closing the phenotype gap, one needs to decide what phenotype(s) to measure and how to interpret the results. Readers of this article may also get a sense that there are several alternative meanings of “stress resistance” (this is perhaps most pronounced for drought where the term “drought tolerance” is often broadly used for both avoidance of water depletion and true tolerance of low water potentials). Does increased resistance (often referred to as increased tolerance, regardless of whether avoidance mechanisms are involved) mean the ability to better survive a near-lethal stress or the ability to remain more productive during a moderate severity stress? Those approaching plant abiotic stress from an agronomic versus ecophysiology perspective can have differing views of this question. Several types of data indicate that the mechanisms plants use to survive severe stress only partially overlap with mechanisms enabling greater productivity at more moderate stress severities. Thus, there is a need to clearly state and define which view of stress resistance/stress tolerance one is applying when interpreting data. In addition, the challenges of connecting phenotypes observed in the laboratory to real differences in a field environment (and vice versa the challenge of achieving a mechanistic understanding of quantitative traits related to yield and stress resistance) demand a certain level of circumspection from those working at all levels of plant stress biology.

For cellular studies of abiotic stresses such as drought and temperature stress, we have a particularly challenging question of how plants perceive the stress at the molecular scale. Such abiotic stress perception does not follow the familiar receptor–ligand paradigm many of us learned in biochemistry class (back when it was enough just to know the answers to the instructor’s questions). Without knowing the beginning, how the plant perceives a change in its environment, it is much harder to understand the downstream responses at any scale. One emerging area of interest is the plasma membrane, and its interfaces with the cell wall and cytoskeleton, as a logical place for plants to sense environmental signals and control water and solute transport while also initiating down-stream signaling and induction of signaling intermediates such as abscisic acid (ABA). Yet we know relatively little about how the cell wall–plasma membrane–cytoskeleton interface acts in stress sensing and signaling and what the key molecular players in stress sensing are.

If we scale up to the whole plant level, there are long-standing questions about how plants control the movement of water through the soil–plant–atmosphere continuum and how stress responses may be coordinated across different tissues. For drought stress, it is sometimes assumed that the initial sensing events occur in roots because they are directly exposed to drying soil. But this need not be the case as the whole plant is hydraulically connected and changes in water potential at the root will be quickly propagated through the plant. Conversely, one could make an equally logical hypothesis that water limitation is first sensed in leaves as these are the site of water loss to the atmosphere and the site where stomata must quickly respond to restrict water loss when water supply from the roots and vascular system is disrupted. There is also a question of whether there are nonhydraulic, chemical signals that move from root to shoot, presumably in the xylem, to communicate a change in water status. The type of signal that this could be remains uncertain, although peptide signaling has recently received increased attention (Takahashi et al., 2018; Reichardt et al., 2020). These hypotheses of root versus shoot sensing and nonhydraulic signaling versus hydraulic signaling are not mutually exclusive and it seems likely that something as important for the plant as sensing changes in water availability (or changes in salinity, temperature, or CO_2_ levels) is likely to have multiple mechanisms which operate in both distinct and overlapping ways. Under severe water limitation, disruption of water transport via xylem embolism becomes more likely and there is ongoing debate on how and when (or whether) refilling of xylem and recovery of hydraulic conductance can occur. There is also debate on how much loss of vascular function and hydraulic conductivity is lethal, either to specific tissue which becomes cut off from water supply or to the plant as a whole.

The concept of tradeoffs, and how they are regulated, is also a recurring theme of plant stress research. The concept of a “growth-versus-defense” trade-off is now frequently mentioned in molecular studies (including sometimes in studies that have data for only one side of the proposed tradeoff). While the “defense” side of the tradeoff may refer to pathogen defenses, which often cause obvious disruption of plant growth, this has gradually broadened to include many types of stress responses that may, either directly or indirectly, affect growth. Another trade-off example is the concept that plants can take a “water spender” strategy of maximizing carbon acquisition even at the cost of high water use versus a “water saver” strategy of restricting water use and acquiring less carbon but maximizing water use efficiency (WUE; amount of carbon acquired per unit of water lost via transpiration). Which strategy is better for a species depends not only on the environment but also on which other plants share that environment. Saving water in the soil for later is less effective if your neighbor spends it first. An important question for research is whether these trade-offs, or other ecologically important tradeoffs, can be broken (high WUE and rapid growth, for example).

In the sections below, we consider these and related questions with the hope that other researchers will be informed and motivated to add, and answer, many other questions about plant abiotic stress that we do not yet know to exist.

## Can plasticity in traits beneficial in both wet and dry soils be recognized and used to limit crop yield loss?

### (By Julia Bailey-Serres)

Increased climate variability is responsible for excessive wet and dry soil conditions that affect irrigated and rain-fed agriculture. From this, there arises a question of whether there are genes and traits, or sets of genes and traits, associated with greater plant resilience in both of these extreme soil environments. One can find support for this notion in species that thrive in ephemeral wetlands. These possess constitutive adaptive traits or display plastic acclimation strategies that facilitate survival in areas that undergo a seasonal rise and ebb of the water table that inundates root systems and can partially or completely submerge aerial tissues. More often than not, wet and dry cycles occur in succession, necessitating traits that are plastic or beneficial under both extremes. Few crops withstand water-saturated soil (waterlogging), let alone submergence for more than several days. Rice is an exception, surviving by accelerating or dampening underwater growth. Flooding escape of seedlings is aided by ANAEROBIC GERMINATION 1, encoding a trehalose 6-phosphate phosphatase, that increases sink strength of the snorkel-like coleoptile, allowing the germinating seedling access to air (Kretzschmar et al., 2015). Deepwater rice can outgrow a seasonal rise in paddy depth of over 3 m. Within submerged stems, ethylene activates a gene suite (SNORKEL1/2, SEMIDWARF1, and ACCELERATOR1) that amplifies cell division at stem node meristems and subsequent internode elongation (Hattori et al., 2009; Kuroha et al., 2018; Nagai et al., 2020). In contrast to this adaptive strategy, the submergence tolerance regulator SUBMERGENCE1A (SUB1A), encoding an ethylene-responsive transcriptional regulator factor subfamily VII (ERF-VII), limits the exhaustion of leaf carbohydrate in leaf elongation (Fukao et al., 2006) and minimizes postsubmergence ROS and water deficit (Fukao et al., 2011). This transient tolerance protects semi-dwarf paddy rice from deep but short-term flash floods. While these studies focus on rice, there are species in all major crop families that are adapted to transient wet zones (*Oryza* and *Zea* in the Poaceae, *Lotus* in the Fabaceae, *Solanum dulcamara* in the Solanaceae, and *Rorippa* in the Brassicaceae). These, along with rice and flooding tolerant Arabidopsis (*Arabidopsis thaliana*), provide insight into plastic survival strategies lost during crop domestication or selection for production agriculture.

Roots perceive subtle changes in soil moisture including flooding, which restricts diffusion of gases, elevating ethylene and depleting O_2_. Can the discovery of regulatory mechanisms accelerate improvement of waterlogging resilience in crops without a yield penalty? Might this be accomplished even if flooding is followed by water deficit?

Let us consider root system traits that are associated with survival of waterlogged and anaerobic soils. When roots of diverse crops (e.g. rice, tomato, and Medicago) become O_2_-deprived, a conserved low-O_2_ gene regulatory network (GRN) is activated by SUB1A-like ERFs that are stabilized as O_2_ levels fall (van Dongen and Licausi, 2015; Reynoso et al., 2019). The genes with conserved ERF-VII cis-regulation encode enzymes of anaerobic metabolism, turnover of ERF-VIIs upon reoxygenation, and ABA perception. Without aeration, as in root meristems of fully submerged rice, DNA synthesis and the cell cycle are attenuated until shoots are re-aerated (Reynoso et al., 2022).

Aeration in flooded soils is enhanced by conditional proliferation of roots with a shallow angle. In rice, this is augmented by loss-of-function of *SOIL SURFACE ROOTING1*, a homolog of *DEEPER ROOTING1* (*DRO1*) (Kitomi et al., 2020), limiting auxin-mediated gravitropism. Also important are shoot-borne (adventitious) roots that emerge near the air–water interface, capturing dissolved O_2_ and nutrients of floodwaters (Lin et al., 2021). Their emergence is auxin-mediated, triggered by ethylene, and localized production of reactive oxygen species (ROS). Aeration is enhanced by internal or external passageways called aerenchyma that provide a low resistance path for the diffusion of O_2_ and other gasses between aerated shoots and waterlogged roots (Pedersen et al., 2021). Aerenchyma form within the cortex of rice and maize roots through ethylene- and ROS-triggered programmed cell death (He et al., 1996; Yamauchi et al., 2016, 2017); auxin signaling is also implicated in rice (Yamauchi et al., 2019). Another characteristic of waterlogged roots is the accumulation of suberin lamellae, a layered polyester of poly(phenolic) and poly(aliphatic) fatty acids, in the apoplasm of the exterior side of the outermost cortical layer (exodermis) or periderm (epidermis of older roots and stems) (Pedersen et al., 2021). Accumulation of the suberin lamellae is mediated by ABA in rice (Shiono et al., 2022). The extension of this gas and water impermeable barrier toward the root tip limits the outward diffusion of O_2_ en route to root meristems.

Aeration traits can be constitutive, as observed in paddy weeds (i.e. *Echinochloa* species; Ejiri and Shiono, 2019) and Amazonian *Oryzae* (Ejiri et al., 2020), or induced by waterlogging or prolonged (stagnant) flooding. Both cortical aerenchyma and exodermal suberin are constitutive in the wetland teosinte *Zea nicaraguensis*, but induced by ethylene in domesticated maize cultivars (Abiko et al., 2012). The mapping and marker-assisted breeding of genetic determinants of these plastic traits are important for crop improvement. Promising examples include a locus associated with adventitious rooting upon waterlogging in soybean that enhances yield stability (Ye et al., 2018) and loci determining aeration traits in teosinte that have been pyramided into maize cultivars (Mano and Omori, 2013; Mano and Nakazono, 2021).

**Figure 1:**
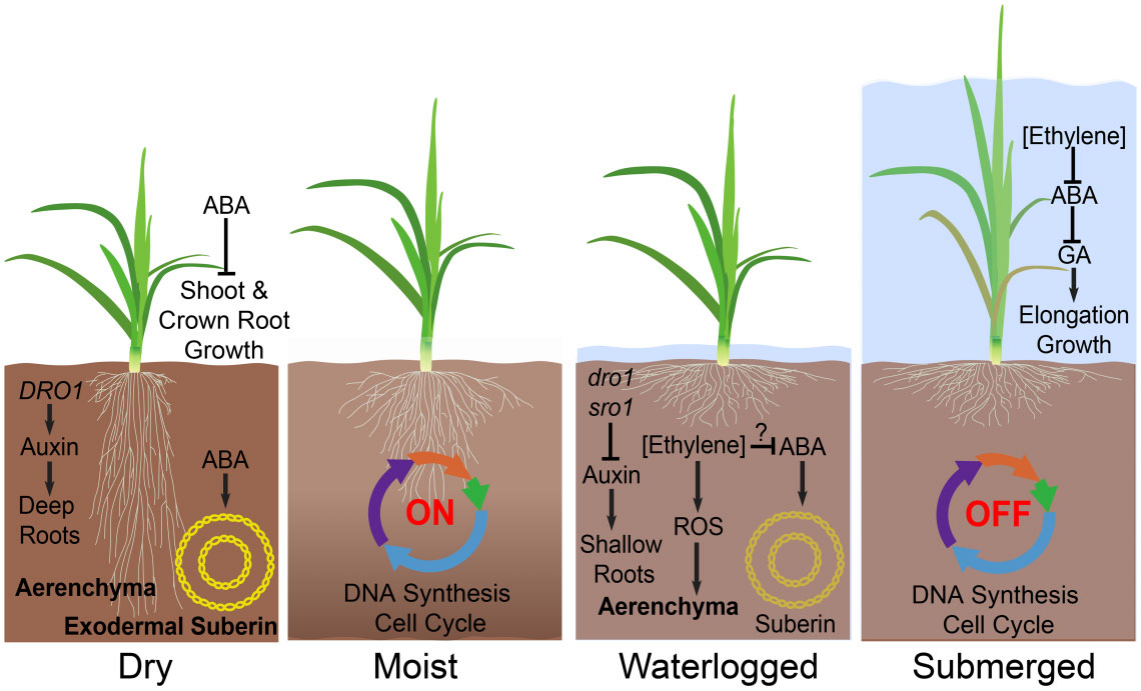
Characteristics of shoot and root growth plasticity associated with variation in soil moisture using semi-dwarf rice as the model. Roots in dry soils grow toward water reserves and limit water loss by deposition of a pronounced suberin barrier, particularly surrounding the exterior side of the exodermis. In moist soils, root architecture is more compact, crown root initiation and elongation are active, and exodermal suberization is limited. Waterlogged roots containing aerenchyma proliferate near the soil surface and exodermal suberin prevents oxygen loss by radial diffusion. In fully submerged plants, cell division activity in root systems is rapidly switched off and leaf elongation is enhanced, promoting escape to the air. Key phytohormones, their relationships and genes mentioned in the main text are shown.

Can root aeration traits be beneficial in dry soils? Water deficit also activates the formation of aerenchyma, a strategy that purportedly reduces metabolic costs of deep water-seeking roots (Lynch, 2015). It also reinforces the exodermal suberin barrier to limit water loss by diffusion between the root tip zone and distal regions. In contrast to the shallow-angle roots of wetland rice, constitutive deep rooting is characteristic of drought-resilient crops. In upland rice, a functional *DRO1* allele promotes auxin-mediated gravitropism resulting in a deep root angle (Uga et al., 2013). Yet plants with shallow root systems that allow greater access to phosphate, nitrogen and other nutrients can display hydrotropism (growth toward moisture through gradient sensing) and hydropatterning (Bao et al., 2014; Robbins and Dinneny, 2018; Lind et al., 2021), discerned as auxin-mediated but ABA-limited lateral branching on the moist side of a root in Arabidopsis (Orosa-Puente et al., 2018). These traits, along with the prioritization of elongation of established crown (nodal) or other roots with access to moisture, may provide sufficient plasticity for survival of a wet to dry transition (Figure 1). But a challenge may be whether spatial or temporal perception of ABA promotes or inhibits root growth in the specific environmental context and species. Another consideration is the consequence of waterlogging on beneficial plant–microbe interactions. Rhizobial nitrogen fixation in legumes and mycorrhizal fungal interactions are limited by waterlogging, yet both recover as soils dry (Justino and Sodek, 2013; Groen et al., 2022).

Comparative genomics, systems biology at the tissue and cell level, and genome editing have expanded opportunities to address these questions to overcome the urgent challenge to increase water extreme resilience in crops.

## How do plants sense and communicate water deficit?

### (By Alexander Christmann and Erwin Grill)

Homeostasis of water status is a major challenge for plants. The gradient of water potential (*ψ*_w_) from the soil to the plant and subsequently to the atmosphere generates a hydraulic continuum that efficiently mobilizes soil-borne water via stomata transpiration into the air. Water availability and the water-conducting capacity of plants impose constraints on this water flux. Regulatory processes governing long-term adjustments and stomata responses to changes in water status are well understood on a molecular level and involve the phytohormone ABA (Yoshida et al., 2019a, 2019b; Zhang et al., 2022). However, the molecular components of sensing water deficit and communicating these cues within the plant remain largely speculative.

### Effects of water deficit

**Figure 2:**
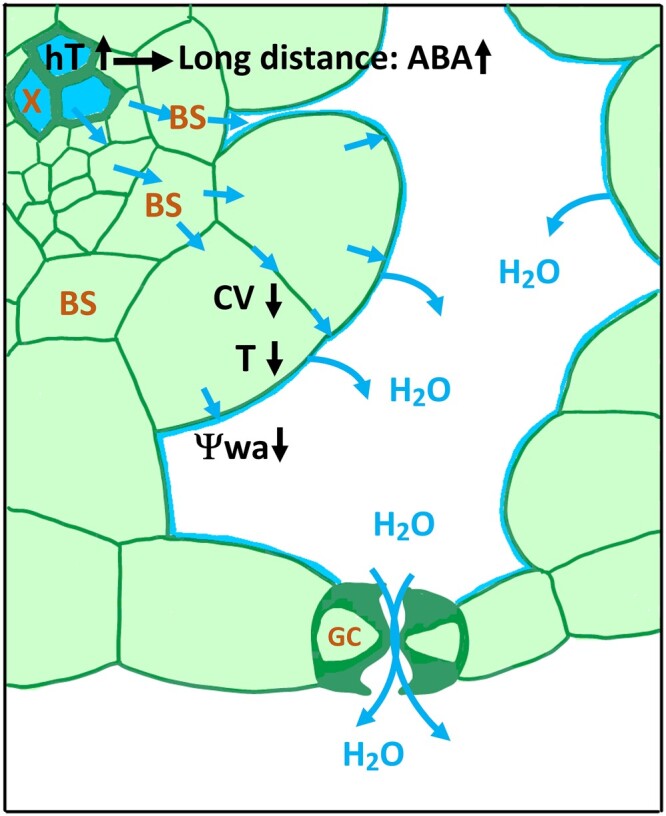
Water flux from the xylem vessel to the evaporation site in a schematic leaf cross section. Water transported through the xylem (X) of a small leaf vein passes through bundle sheath (BS) cells to mesophyll and epidermal cells, which surround guard cells (GCs), the sites of transpiration. GCs are symplastically isolated (have no plasmodesmata) and water supply occurs from the apoplast. Transpiration exceeding water replenishing at the evaporation sites results in a reduced water potential of the apoplastic fluid (*ψ*_wa_). This key event triggers a number of changes: a reduction of turgor (*T*) and cell volume within the mesophyll, and an increase in the hydraulic tension (hT) within the vasculature. The change in hT serves as fast long-distance signal and there might be parallels with mechanical sensing and relay of touch and wounding. The hydraulic signal is converted into the chemical signal ABA to adjust water conductance and stomatal aperture. Mechano-sensitive components are likely involved in all cell types but have not been unequivocally identified.

Uptake of soil water by plants requires a root ψ_w_ more negative than the surrounding soil, which is achieved predominantly by osmotic adjustment and negative hydraulic pressure due to transpiration. A negative water balance (i.e. water uptake lower than its release by transpiration) immediately changes a number of parameters within the plant. In the substomatal cavity, the major evaporation site, *ψ*_w_ of the apoplastic fluid becomes more negative, causing neighboring cells to lose water, and consequently causing their turgor and volume to decrease (Figure 2). Simultaneously, the hydraulic tension increases within the xylem. These water deficit-induced changes are relayed within the tissue and, depending on the extent of water loss and water capacitance of the plant tissue, possibly to other organs. Changes in hydraulic parameters serve as a fast long-distance signal (>40 cm min^−1^) (Christmann et al., 2013). Suppression of the hydraulic signal from desiccating roots blocked the ABA-induced leaf response, that is, stomatal closure and ABA-dependent gene expression (Christmann et al., 2007). Reduction of stomatal aperture by ABA signaling readjusts the water balance by lowering transpiration. Stomatal responses to high water vapour deficit are regulated in a largely guard cell-autonomous manner but with pavement cell–guard cell interaction. Signaling elements of this response include several protein kinases, Raf-like proteins, and the receptor-like kinase GHR1, which act upstream of the ABA response mediator Open Stomata 1 (OST1) (Hsu et al., 2021). Improved water uptake via increases in root hydraulic conductance (Maurel et al., 2016) and osmotic adjustment recover leaf gas exchange at the expense of increasingly negative plant *ψ*_w_. These adjustments are incremental and occur even under nonstress water conditions.

### How is the change in *ψ*_w_ in the plant perceived?

Changes of tissue *ψ*_w_ relative to its surroundings affect cellular water flux, osmolarity, and mechanical forces including turgor acting on the cell wall, biomembranes (e.g. plasma membrane, tonoplast, and endomembranes), and the cytoskeleton. There is mounting evidence for a mechano-sensitive mechanism that detects these changes. Classical paradigms for mechano-sensitive and *ψ*_w_ change-mediated plant responses are touch-induced leaf closure of the Venus flytrap and tendril coiling of Bryonia that are very sensitive to external force, at least as sensitive as human touch. (Klusener et al., 1995; Escalante-Perez et al., 2011) Thus, touch-sensitive signaling offers a good model to conceptualize the types of mechanisms that could be involved in low *ψ*_w_ perception and initial signaling.

The Venus flytrap uses multicellular trigger hairs to sense prey by converting mechanical forces exerted by the insect into a turgor-driven snap. The response to insect touch is relayed to neighboring cells by electrical signaling involving a calcium (Ca^2+^) wave within the leaf lobe (Suda et al., 2020). The sensory cells of the trigger hair are enriched in ion channels associated with mechano-perception and Ca^2+^ entry (Iosip et al., 2020; Procko et al., 2021). These include homologs of the Arabidopsis mechano-sensitive-like channel 10 (MSL10) (Basu and Haswell, 2020), the glutamate receptor Ca^2+^ channel GLR3.6 and hyperosmolality-induced [Ca^2+^]-increase (OSCA)-family channels. Members of the OSCA family are mechanically activated and ion nonselective (Murthy et al., 2018). Touch-induced depolarization of the sensory cells is mediated by MSL10 and initiates action potentials that propagate via GLR3.6 and OSCAs. MSL10 and GLR3.6 are known components of electrical signal propagation upon wounding (Toyota et al., 2018; Farmer et al., 2020; Moe-Lange et al., 2021). In the case of wounding, hydraulic pressure waves are initiated that propagate through the xylem and trigger an electrical and Ca^2+^ wave in the vasculature. The waves are relayed (10 cm min^−1^; Farmer et al., 2020) by MSL10 and may require downstream-acting GLRs localized to endomembranes, namely the phloem-expressed GLR3.3 and GLR3.6, which are also highly expressed in xylem-contacting cells (Nguyen et al., 2018; Moe-Lange et al., 2021). Hence, mechano-sensing in the Venus flytrap and wounding response utilize many of the same molecular components and also share a signal propagation mechanism that includes a wave of increased intracellular Ca^2+^. Endomembrane compartments also play a critical role in Ca^2+^ release during mechano-stimulated responses (Klusener et al., 1995). Mechanical forces acting from outside the cell can be relayed to these intracellular cell compartments by the cytoskeleton and cytoskeleton-associated proteins (Bhaskara et al., 2017; Hamant et al., 2019) or by the loss of water causing osmotic disequilibrium between intracellular compartments which may activate stretch related sensing on intracellular membranes in addition to the plasma membrane. The involvement of endomembrane signaling in stress response is also indicated by observations that loss of the chloroplast-localized mechanosensitive channels MSL2 and MSL3 leads to osmotic imbalance between chloroplast and cytosol and constitutive activation of low *ψ*_w_ response in unstressed plants (Wilson et al., 2014).

The electrical wave-induced cell depolarization involves additional components shared with ABA responses such as voltage-dependent anion and cation channels, NADPH-oxidase, and the proton ATPase driving re-polarization (Farmer et al., 2020; Iosip et al., 2020). While wounding results in a sudden relaxation of the hydraulic tension at the severed xylem, water deficit increases this tension. The increased xylem tension translates into a stronger pulling force acting on the xylem-contacting cells. A sudden change of this force might distort and stretch domains at the plasma membrane. Pulling forces of −0.1 bar resulted in half-maximum MSL10 activation in the Venus flytrap (Procko et al., 2021) and even relatively mild water deficit could be expected to generate similar or stronger forces. Touch- and wound-activated responses also induce chemical signals including oxo-phytodienoic acid and jasmonate for subsequent phytohormone signaling (Escalante-Perez et al., 2011; Farmer et al., 2020). We currently do not know the extent to which touch- and wound-induced signaling mechanisms overlap with the mechanisms used to detect and respond to water-deficit. If such mechanisms are involved in water deficit signaling they would be expected to induce ABA accumulation as a key signaling factor to turn on further downstream stress responses.

Genetic screens for Arabidopsis mutants impaired in regulating ABA-responsive reporter expression under hyperosmotic stress have failed so far in identifying water-deficit sensing receptors (Wang et al., 2011; personal experience). Genetic and functional redundancy provides an explanation, in which receptors act in parallel pathways that converge on ABA. This might be the case considering the multiple cellular effects of leaf transpiration exceeding water uptake. Use of Arabidopsis natural variation in low *ψ*_w_-induced ABA accumulation identified candidate loci affecting ABA accumulation (Kalladan et al., 2017); however, the possible role of these candidate loci in stress-signaling needs to be validated and further studied. The increased tension of xylem water may activate MSL-type channels of contact cells or mechanical force-sensing Ca^2+^ channels as part of long-distance communication. These contact cells and the surrounding parenchyma of the vasculature play a prominent role as specific sites of ABA biosynthesis (Endo et al., 2008). Cells lining the substomatal evaporation sites are particularly challenged with water efflux and turgor decrease. A decrease in cell volume and the concomitant reduction in plasma membrane surface (Sack et al., 2018) could be sensed similar to yeast target of rapamycin (TOR) complex2 that balances plasma membrane constraints with membrane lipid level (Riggi et al., 2019). Turgor-sensing and—controlling mechanisms need to be activated to re-establish water balance. However, bona fide turgor sensors of plants are not known yet. In yeast, the histidine-kinase SLN1 senses turgor and controls the hyperosmolarity response (Reiser et al., 2003). Interestingly, several structurally related histidine kinases of Arabidopsis, including AHK1 and the cytokinin receptors AHK2, AHK3, and AHK4, can complement the turgor-sensing function of SLN1 either alone or in the presence of cytokinin for AHK4 (Reiser et al., 2003; Tran et al., 2007). However, it is unclear whether AHKs act as water stress sensors in plants (Kumar et al., 2013). RAF-like protein kinases involved in osmotic adjustments are promising candidates for downstream-acting signaling components (Lin et al., 2020; Soma et al., 2020).

Components that maintain or monitor cell wall integrity are critical in stressful conditions exerted by high turgor pressures. Such components include pectate lyase (Chen et al., 2021) and several plasma membrane-localized protein kinases like FERONIA, required to avoid root cell burst in response to salt stress (Feng et al., 2018), CrRLK1L/BUPS1 (Zhou et al., 2021), THESEUS (Bacete et al., 2022), and STRUBBELIG (Chaudhary et al., 2021).

In summary, water deficit responses are activated during increasing transpirational demand to restore plant water homeostasis and sustain photosynthesis. Understanding how plants sense and communicate water deficit on a molecular level provides a promising tool to increase WUE without yield penalty needed for crops of the future (Yang et al., 2019).

## How, where, and when are water deficit signals integrated during floral transition?

### (By Lucio Conti)

**Figure 3:**
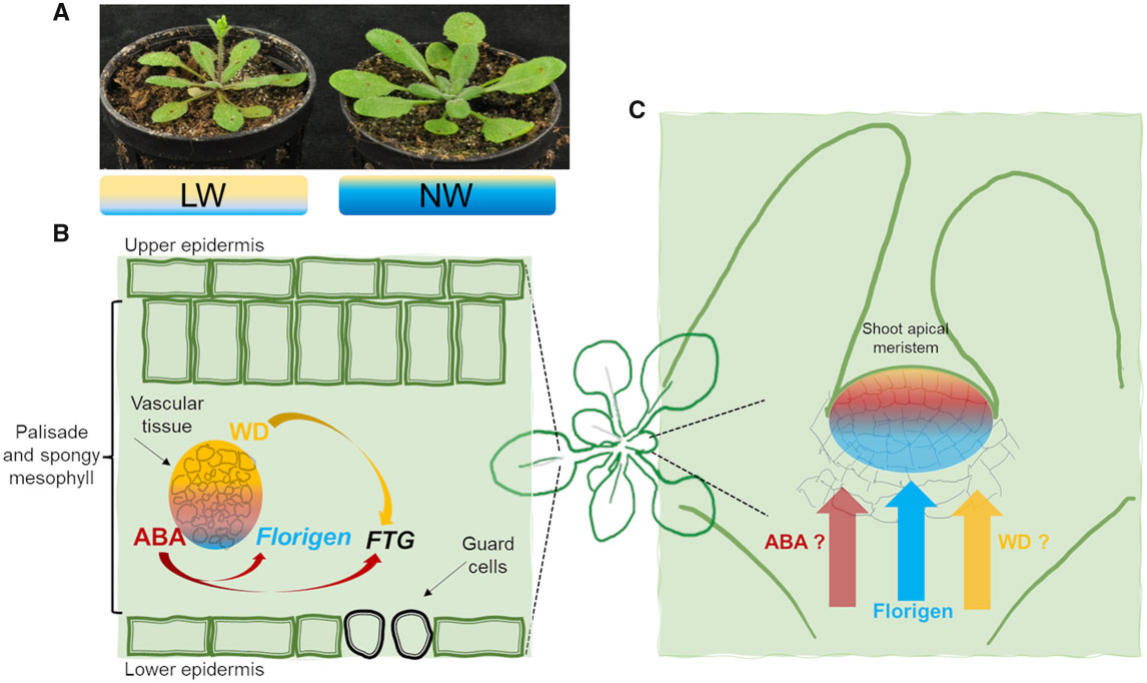
Known and uncharacterized signals causing DE and their sites of action. A, wild-type Arabidopsis plant photographed at ∼3 weeks from germination. Plants were grown under normal or low-watering (NW and LW, respectively) regimes. LW plants exhibit DE. B, Cartoon outlining a leaf cross-section. Water deficit signals (WD) are transmitted through the vasculature to stimulate ABA production. ABA positively contributes to florigen transcriptional activation in leaves in response to water deficit. ABA and WD promote expression of many other flowering time genes regulating guard cell activity and drought tolerance traits. C, Besides florigen proteins, different signals converge at the shoot apical meristem to relay water deficit information and regulate floral transition accordingly. Despite the ability of the shoot meristem cells to integrate these cues, it is still unclear how water deficit signals (WD) can reach the apex and if significant ABA translocation occurs.

Plant physiology textbooks emphasize how plants are continuously challenged by their surrounding environment and how this triggers developmental adjustments. But what happens if the environment deteriorates to the point of threatening survival? Stressors like water deficit can pose such a challenge for plants and one survival strategy relies on the high flexibility of the flowering program. The switch to flowering is a crucial decision for plants, determining the cessation of the vegetative phase and initiation of reproductive development. It occurs at the shoot apex upon receipt of environmental and endogenous signals and precedes shoot elongation, specification, and outgrowth of floral organs. Depending on the ecological context appropriate timing of the floral transition positively influences inflorescence growth, architecture, and the number of flowers produced (as these processes rely on carbon assimilation from vegetative leaves). Several studies describe the rapid natural and artificial selection for early flowering phenotypes to evade harsh summer drought scenarios (Franks, 2011; Kenney et al., 2014; Monroe et al., 2018; Groen et al., 2020), a strategy referred to as drought escape (DE). DE permits the completion of the life cycle before water deficit conditions become extreme, even if at the expense of fitness. Flowering time regulation is also extremely plastic in the face of unpredictable environmental constraints (Blackman, 2017). Similarly, DE can also be adaptive (Figure 3A) which means that upon experiencing water deficit, some species can activate an earlier floral transition, although significant variations in genotype by water deficit interaction exist (Franks, 2011; Kenney et al., 2014).

Where does DE originate? Flowering is regulated through a network of genes that perceive environmental/endogenous signals and genes that integrate these signals to regulate floral induction. Florigen genes are important integrators expressed in the vascular tissue of vegetative leaves. They encode a class of small globular proteins that move long-distance through phloem vessels at the shoot apex to cause transcriptional reprogramming of meristem cells and floral specification of new primordia. While florigen’s transcriptional activation usually occurs upon perception of critical daylengths, studies in Arabidopsis, rice, and tomato further point to the contribution of water deficit signals (Riboni et al., 2013; Du et al., 2018; Chong et al., 2022). Transcriptional upregulation of florigen genes in response to water deficit appears to be necessary and sufficient to cause DE in these species, despite their evolutionary separation. The phytohormone ABA emerges as a conserved molecule regulating florigen expression, although different mechanisms are involved (Figure 3B). In tomato, ABA stimulates phosphorylation and subsequent nuclear shuttling of the transcription factor VASCULAR PLANT ONE-ZINC FINGER 1 to activate the florigen SINGLE FLOWER TRUSS (Chong et al., 2022). In Arabidopsis and rice, ABA orchestrates different transcriptional and posttranscriptional mechanisms, with key contribution of a class of ABA-regulated basic leucine-zipper (bZIP) transcription factors that, in Arabidopsis, are incorporated into multimeric protein complexes to activate (indirectly) florigen expression (Yoshida et al., 2014; Hwang et al., 2019).

Are florigens unique in relaying water deficit information at the shoot apex? Considering that ABA is an important driver of DE, its redistribution at the shoot could also influence the flowering process (Figure 3C). Phosphoproteomics studies in Arabidopsis reveal several flowering time regulators as substrates of ABA-activated signaling, suggesting multiple and spatially separate points of control of the floral network (Wang et al., 2013a). Is there ABA translocation at the shoot apex or are shoot meristem cells capable of de novo ABA production in response to water deficit signals? Can ABA directly affect cell fates at this site? These questions remain largely unsolved. In Arabidopsis, ABA biosynthesis occurs in roots and vascular bundles of leaves, largely overlapping with the main sites of florigen expression (Kuromori et al., 2014). ABA is loaded into the phloem to be distributed across different tissues (Kuromori et al., 2018; Daszkowska-Golec, 2022). While measuring ABA cellular concentrations in specific shoot cells remains challenging, there is evidence that florigen proteins can directly control the expression of different ABA signaling genes at the shoot apex (Zhu et al., 2020). ABA and its signaling cascade regulate leaf emergence rates, suggesting a direct influence on meristem cell activity, possibly mediated by regulation of primary metabolism (Yoshida et al., 2019b). These findings could set the stage for a better understanding of ABA-regulated cell fate reprogramming in response to, or in parallel with, florigen mobilization at the shoot in response to water deficit. Additionally, this would stimulate further questions about the role of ABA accumulation and signaling in flowering time regulation under optimal irrigation conditions and its conservation across species. For example, reducing ABA sensitivity of rice plants by mutations in a clade of ABA receptors causes late flowering, a phenotype that is not observed in Arabidopsis (Miao et al., 2018).

Many questions remain concerning the number of signals elicited in response to water deficit, their integration in reproductive development, and the role of flowering time genes in conferring drought protection (Figure 3, B and C). Water deficit-stimulated ABA production inhibits shoot growth, thereby delaying the appearance of floral structures (LeNoble et al., 2004). Thus, water deficit may lead to uncoupling chronological time to flower (the appearance of floral organs) from floral transition per se. Furthermore, unknown signals can influence shoot meristem function, flowering time, and florigen expression, depending on the level of water deficit imposed (Galbiati et al., 2016; Du et al., 2018). How cells can distinguish between varying levels of water deficit is unclear. ABA also activates negative regulators of flowering, including FLOWERING LOCUS C (FLC) in Arabidopsis (Wang et al., 2013b). FLC contributes to delaying flowering, which is most apparent when water deficit occurs under noninductive daylength conditions (i.e. when florigen expression is repressed) (Riboni et al., 2013). Because FLC antagonizes the expression of multiple floral genes in leaves and at the shoot apical meristem, its activation may be required to curb excess florigen signaling and finetune inflorescence development according to stress intensity. Concurrent regulation of antagonistic flowering mechanisms may also generally impact drought tolerance traits. For example, plants carrying functional alleles of FLC and its upstream regulator FRIGIDA (FRI), display strong reductions in water use (Mckay et al., 2003). SHORT VEGETATIVE PHASE, a floral repressor related to FLC, promotes ABA accumulation (Wang et al., 2018b). In contrast, the florigen gene FLOWERING LOCUS T (FT) regulates stomatal opening, favoring transpiration (Kinoshita et al., 2011). Considering the importance of the duration of crop cycles on yield and the role of ABA in reducing water loss, identification of DE molecular mechanisms and their natural genetic variations could offer targeted strategies to balance flowering time and drought tolerance traits.

## How do we incorporate plant diversity into our molecular understanding of environmental stress adaptation?

### (By José R. Dinneny)

It is an obvious fact, but worth reiterating, that plants have evolved to occupy nearly every environment on the earth’s surface (Corlett, 2016). Furthermore, through agriculture, humans have bred plants that are now cultivated across 12.6% of the total terrestrial landscape (Global Cropland Extent Product at 30 m (GCEP30) (Thenkabail et al., 2021). The ability of plants to occupy this breadth of environments involved the evolution or breeding of plant physiological mechanisms to meet the diverse environmental challenges that are faced in each ecosystem and agricultural management system. Despite this clear abundance of physiological diversity, the majority of mechanistic research in plants is still focused on a small collection of stress-sensitive model systems. This is not to say that such discoveries are unimportant or limited in impact, however, we have a patchy understanding as to whether such studies will identify broadly relevant principles, or rather species-specific details. Addressing the question of how plant–environmental responses are diversified across the kingdom will provide insight into the major innovations plants have evolved to survive in different environments and will also inform strategies for introducing such mechanisms into a broader range of crop plants.

Much of the past emphasis on investing in molecular genetic model systems such as Arabidopsis was based on the historic limitation in the availability of genomic resources. Furthermore, these concerted efforts established a critical mass of researchers focused on determining a baseline understanding of plant molecular biology (Provart et al., 2016); however, this is no longer applicable. The Kew Royal Botanic Gardens recently estimated that ∼374,000 plant species have been discovered (Christenhusz and Byng, 2016) and recent efforts have led to genome sequences being available for over 350 species. Botanic gardens have made efforts to sequence the genomes of their collections and the Ruili Botanic Gardens in China has done so for 689 species (Liu et al., 2019). The 10,000 Plant Genomes Project run by the Beijing Genome Institute promises to expand this list of available sequences far beyond what is currently available (https://db.cngb.org/datamart/plant/DATApla1/). Furthermore, transcriptomic profiling of species across the green plant lineage (Leebens-Mack et al., 2019), or occurring in specific ecological niches, such as the Atacama Desert (Eshel et al., 2021), has clarified phylogenetic relationships and illuminated physiological adaptations of plants to arid climates. In short, the book of life for plants is being revealed at an extraordinary pace.

Despite this rapid progress, the pace of studies that functionally explore this glut of genomic data has not kept pace. The bottlenecks that limit our ability to functionalize genome sequences and discover the molecular mechanisms governing adaptations to the environment are three-fold. The first limitation is our understanding of the diverse physiological adaptations that plants use to survive environmental challenges. The second is the paucity of methods available in nonmodel plants to enable the functional characterization of a plant’s genomic sequence. Finally, limitations in the ability to manipulate the genomes of a diverse array of species through genetic engineering prevent hypothesis testing about genotype–phenotype relationships and the application of this knowledge.

The lack of understanding of the diversity of physiological adaptations to the environment is particularly apparent for the root system, which by its nature has remained hidden behind a veil of soil. Roots can be considered the sustainability organ system (Lynch, 2007). They function to provide the plant anchorage and prevent displacement from a fixed position in the ground (Hostetler et al., 2021b), but other processes are also relevant. Roots are the major conduit for the absorption of water and nutrients and roots engage in a metabolic bartering system with soil microbes, which facilitates nutrient uptake in exchange for the products of photosynthesis (Fitzpatrick et al., 2018). Plants also communicate with each other through their roots and this can affect the density and diversity of local communities (Mommer et al., 2016). Despite these varied functions, very little molecular insight into how roots perform these functions has been described beyond in a few model species. Furthermore, of the tissues and cell types that are thought to compose most roots, little is understood as to whether plant species have evolved innovations in cellular function that allow them to survive in the vast array of soil types and terrains on earth. Innovations in the preparation of plant tissue for light-based microscopy, such as ClearSee (Ursache et al., 2018), and in the use of other imaging modalities, such as microscopic computed tomography (microCT) (Mairhofer et al., 2011; Morris et al., 2017), have opened up opportunities for the quantitative exploration of diverse plant anatomical structures and of root systems grown in soil.

**Figure 4:**
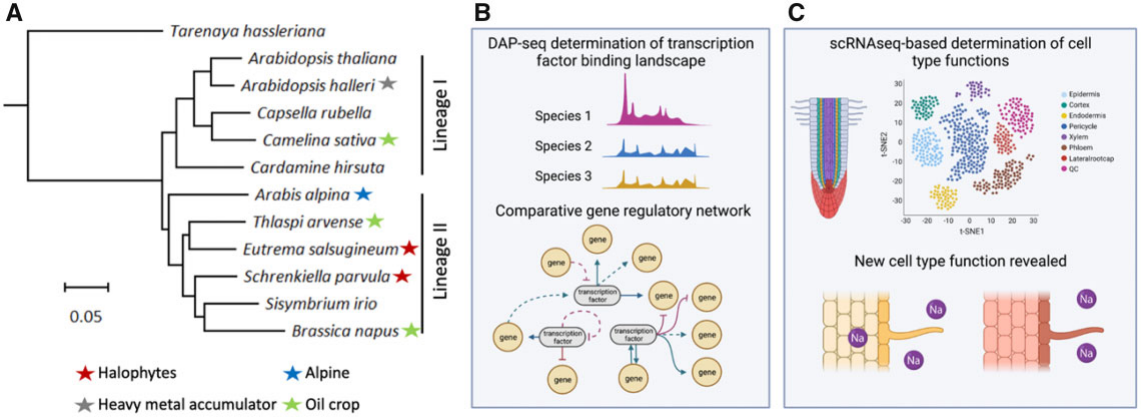
The Brassicaceae family contains species with diverse physiological traits and agronomic value. A, Phylogeny of species in the Brassicaceae family with sequenced genomes. Stars indicate different physiological or agronomic properties of interest. Figure courtesy of Dong-Ha Oh. B, DAP-seq facilitates the rapid determination of transcription factor–genome interaction landscapes. GRN architecture can be compared between species to identify rewiring events. C, scRNA-seq enables cell-type functions to be explored between species and for new functions to be uncovered.

Specific plant families such as the Brassicaceae (Koenig and Weigel, 2015), Solanaceae (Fernandez-Pozo et al., 2015), and Poaceae (Buell, 2009) have emerged as models for comparative genomic studies. Encompassing ∼3,630 species, the Brassicaceae family is home to plant species used in agriculture including oilseed crops *Brassica napus* (canola) and *Camelina sativa*, salt-tolerant halophytes species *Eutrema salsugineum* and *Schrenkiella parvula*, and the well-characterized model molecular-genetic plant Arabidopsis (Figure 4A). Arabidopsis provides a nested model system within the Brassicaceae family for exploring the diversification of stress responses. The sequencing of over 1,000 accessions facilitated the identification of genetic loci under selection, and identified the ABA signaling pathway as being important (1001 Genomes Consortium, 2016). ABA, which is induced under drought and salinity stress (Cutler et al., 2010), suppresses root growth in Arabidopsis, particularly at concentrations above 1 µM, while in other species like *S. parvula*, an extremophyte plant living at the edge of a hyper-saline lake, ABA accelerates growth (Sun et al., 2022). These data suggest that even for well-characterized signaling pathways, diametric changes in response to ABA are possible. Further exploration of the diversification of stress response pathways will help to reveal the principles behind the tuning of such pathways during evolution.

Methodological innovations are beginning to provide functional insight into nonmodel plants (Figure 4, B and C). Single-cell RNA sequencing (scRNA-seq) now allows cell type-specific transcriptional profiles to be obtained from a diverse range of species (Tarashansky et al., 2021). This provides insight into the diversification of cell type functions and the potential discovery of new cell types. DNA affinity purification and sequencing (DAP-seq), which allows for the in vitro reconstitution of transcription factor–genome interactions (O’Malley et al., 2016), enables the determination of GRNs in nonmodel species (Sun et al., 2022). Comparisons of GRN architecture between related species provide a means of understanding how the evolution of genomic sequence leads to the rewiring of GRNs and the regulation of downstream physiological processes important for stress acclimation. These advances, together with improvements in gene editing and plant transformation (Nadakuduti and Enciso-Rodríguez, 2020; Anjanappa and Gruissem, 2021) dramatically brighten the horizon for studies in nonmodel species.

## How do plants integrate climate signals?

### (By Scott Hayes and Christa Testerink)

#### Integrating stress signals: from field observations to understanding cellular events

Over the past two decades, several canonical environmental signaling pathways have been established. These pathways trace environmental signals from perception to transduction and finally an altered transcriptional and phenotypic response. These advances are a notable achievement. Studying “clean” responses to a single stress has proved a sensible and effective approach to identifying sensors and signaling pathways (Lamers et al., 2020). What is becoming increasingly clear, however, is that many of these signaling pathways are context-dependent. Environmental cues are often transduced through overlapping molecular components, leading to highly contextual molecular responses. While many agronomical and crop science studies have already extensively addressed naturally occurring combinations of environmental stress factors (Rivero et al., 2022), the molecular mechanisms underpinning these interactions are often obscure.

To understand plant growth in complex environments, we must improve our understanding of how different cues are integrated into plant development. In this section, we focus on the cellular pathways governing the integration of abiotic signals, with a focus on water availability and temperature as relevant climate change-related cues, that often coincide (Livneh and Hoerling, 2016). We highlight important factors that need to be taken into consideration when studying signal integration, and we put forward conceptual frameworks through which to study these processes.

#### Genome-wide studies identify interesting patterns

Phenotypic and transcriptomic studies have offered the first clues to the molecular mechanisms involved in signal integration. Pioneering studies found that the transcriptomic and metabolic response of plants treated with both drought and heat stress differed dramatically from when these stresses were applied in isolation (Rizhsky et al., 2004). Proline accumulated in response to drought, but not in response to a combination of drought and heat stress. Instead of proline, sucrose was produced as an osmoprotectant in these conditions (Rizhsky et al., 2004). More in-depth studies, using up to six combinations of five stresses (cold, high light, salinity, heat, or flagellin) found that around 60% of transcriptional responses could not be predicted from the response to single stresses alone (Rasmussen et al., 2013). Sewelam et al. (2014) tested the transcriptional response to salt, mannitol, heat stress, and combinations of the three and also found that transcripts in the combined treatment could not be accounted for by the data for single stresses alone (Sewelam et al., 2014). Most studies into transcriptional signal integration have opted for severe stress levels, and one could argue that the unexplained transcriptional response was caused by tissue damage. However, Prasch and Sonnewald (2013) found that relatively moderate soil drought (30% of field capacity) substantially altered the transcriptional response to warm temperature (32°C/28°C). Several other -omics approaches have expanded our knowledge by documenting the responses to combined abiotic stresses, but few have shed light on the molecular and cellular mechanisms involved in signal integration (Zandalinas et al., 2021). Genome-wide association studies have allowed for the identification of genomic loci important for the interaction between nutrient deficiency signaling and salt stress (Kawa et al., 2016), but these loci remain to be further characterized. And while in-depth phenotypic and transcriptomic analysis has led to the identification of several genes involved in the cross-talk between nutrient deficiency stress (Kellermeier et al., 2014), we still lack a coherent framework for understanding this interaction.

#### Progress toward understanding the mechanisms of signal integration

Several studies have started to probe environmental signal integration on a mechanistic and cellular level. For example, low levels of soil salinity suppress shade avoidance (Hayes et al., 2019). Soil salinity acts through the ABA pathway to suppress brassinosteroid-activated transcription factors, thus limiting shade-induced growth. There have also been significant advances in our understanding of how light and temperature signals are integrated (Hayes et al., 2021). In several Arabidopsis accessions, simulated neighbor shade triggers an increase in petiole elongation at 22°C, but not at 16°C. This striking, temperature-dependent response to shade involves the receptor-like kinase ERECTA (Patel et al., 2013). More recently, it has become clear that shoot temperature perception is heavily integrated with light signaling pathways. Phytochrome and phototropin photoreceptors revert more quickly to their inactive forms at warm temperatures (Legris et al., 2016; Jung et al., 2016a; Fujii et al., 2017), with the result that light signaling is suppressed in these conditions. Additionally, the light-suppressed transcription factor PHYTOCHOME INTERACTING FACTOR 7 (PIF7) exhibits enhanced translation at warm temperatures due to changes in its mRNA structure (Chung et al., 2020). In accordance with the antagonistic relationship between light and warm temperature, shade-avoidance is more aggressive at high ambient temperatures (Romero-Montepaone et al., 2021).

#### The future outlook

Recently, data obtained by Prasch and Sonnewald (2013) were re-analyzed and used by (Azodi et al., 2020) to train machine learning models to predict cis-regulatory elements required for the synergistic response to multiple stresses. Novel approaches using computational models including cellular and functional structural modeling are a promising avenue to capture and predict interactions. In addition, Morales et al. (2021) reported detailed phenotypes of Arabidopsis plants in response to drought and temperature stress in combination with recovery from flooding. Together these studies provide a starting point to investigate the mechanisms underlying consequences of combined stress on plant development and resilience.

**Figure 5:**
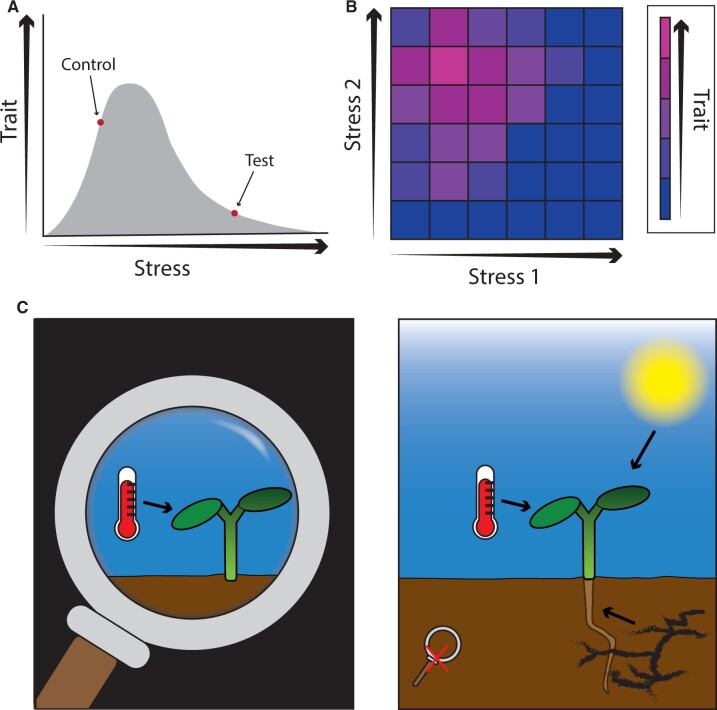
How do plants integrate climate signals? A, Most environmental cues do not have linear effects on plant development. When investigating the molecular basis for a particular cue, it is important to consider that different intensities of the same stress likely act through different molecular pathways. B, Representing gradients of two stresses as a heat map could be a potential way to conceptualize complex interactions. Note that the intensity of Stress 1 affects the way plants respond to the intensity of Stress 2. C, Through taking a step back and studying the interactions between signaling pathways in their relevant environmental context, we will come closer to understanding the molecular basis for plant development in natural environments.

A major limitation of most studies on signal integration is the use of single stress intensities. Many stresses have a nonlinear effect on plant traits, dependent on intensity (Figure 5A). It is likely that different signaling networks act at different intensities of the same stress. When investigating the integration of two stresses, it may be useful to plot traits in a matrix of severity (Figure 5B). Comparing heat map matrices of mutants and wild types may eventually allow us to place signaling networks within specific environmental contexts. It will also highlight environmental contexts in which phenotypes cannot be explained by known developmental regulators and offer potential avenues for future research. Transitioning to gradients of environmental conditions (rather than one, often severe stress condition) will require conceptual and computational advances. It should also be noted that environmental integration is likely to be different for different organs, tissues, or even cell types. Temperature signaling in the roots, for example, can act independently of the light signaling components in the shoot (Bellstaedt et al., 2019; Ludwig et al., 2021). Providing spatial and dose-dependent context of other relevant environmental factors (Figure 5C) will ultimately allow us to understand plant responses to abiotic stress in a realistic, complex environment.

## How do trade-offs impact abiotic stress responses and climate adaptation?

### (By Robert W. Heckman and Thomas E. Juenger)

Trade-offs occur when a phenotype that confers an advantage in one context also confers a disadvantage, whether in the same or a different context (Agrawal, 2020). A classic trade-off is between carbon gain and water loss during photosynthesis: when stomata open to absorb CO_2_, they lose H_2_O (Schulze and Hall, 1982). Numerous ways of mitigating this trade-off have evolved in plants, from minor adaptations like changes in stomatal behavior or development to major innovations like C4 and CAM photosynthesis. From an evolutionary perspective, trade-offs impose costs that constrain the ability of plant populations to evolve in response to selection (Roff and Fairbairn, 2007; Donovan et al., 2011). These constraints may prevent populations from reaching adaptive peaks, particularly in new environments, which can limit species’ ranges and the distribution of habitats suitable for particular crops (Blows and Hoffmann, 2005; Shaw and Etterson, 2012; Dwivedi et al., 2021). Trade-offs can originate from different biological processes, like the specialization of ecotypes in different habitats (Agrawal, 2020). These differences in the origins of trade-offs can impact how plants acclimate, and how plant populations evolve, in response to climate change.

Here, we focus on trade-offs that occur within species as they are most likely to be the subject of ongoing evolution that can drive adaptation. Trade-offs within a species or population can result from pleiotropy and genetic linkage and the degree of standing genetic variation (i.e. genetic architecture) (Saltz et al., 2017). Pleiotropy occurs when the same gene encodes multiple traits; linkage occurs when genes encoding different traits are located in close physical proximity on a chromosome, reducing recombination and resulting in coinheritance (Lynch and Walsh, 1998; Mackay, 2001). In the short term, trade-offs can constrain evolution by linking particular trait combinations and making other trait combinations less likely (Walsh and Blows, 2009). Trade-offs can also be reinforced by correlational selection, which occurs when certain combinations of traits, rather than traits in isolation, are advantageous (Sinervo and Svensson, 2002). Trade-offs can also occur among populations within species. These trade-offs often arise due to local adaptation, which occurs when plants perform better in their home environment than plants from a different environment (Kawecki and Ebert, 2004). Local adaptation results from multiple factors, including antagonistic pleiotropy, where an allele at a genetic locus leads to high relative performance in one environment and low relative performance in a contrasting environment, and conditional neutrality, where an allele confers high (or low) relative performance in one environment and has no impact on performance in a contrasting environment (Anderson et al., 2011).

Understanding how and when trade-offs operate will help biologists to gain the benefits of a trade-off while avoiding the costs. To do this, context is often critical. Trade-offs in allocation to different functions may be hidden when plants grow under benign conditions, such as those in many agronomic and laboratory settings (Roff and Fairbairn, 2007). When plants grow under more natural, stressful conditions, the trade-offs may be exposed, leading to unexpectedly suboptimal performance (MacTavish and Anderson, 2020). This can occur because benign conditions often increase the total resource acquisition by plants. For example, a trade-off between root and shoot biomass is commonly invoked, because when resources are fixed, any allocation to root biomass must come at the expense of allocation to shoot biomass (Shipley and Meziane, 2002). But, in nature, many plants with large root systems also have large shoots, suggesting that no trade-off exists. This failure to detect an allocation trade-off occurs when failing to account for differences in resource acquisition.

Context is also key when considering attributes of wild species in agronomic environments. Often, the characteristics that make wild plants successful become liabilities in crops. In wild plants, many stress-response strategies ensure plant survival via reduced growth rates or stress tolerance strategies, including slow growth, leaf abscission, or early flowering (Fang and Xiong, 2015). In agronomic conditions, where rapid growth and high yield are prized, these stress responses are often maladaptive (Maggio et al., 2018). This may make extremely well-adapted, stress-tolerant wild relatives (or their adaptive strategies) poor targets for domestication or crop improvement. Instead, biologists should re-examine some ideas about what makes plants successful in resource-rich, agronomic environments. For instance, domestication simultaneously increased growth rates and reduced drought tolerance in *Helianthus annuus* (Koziol et al., 2012). Traits like stay-green sorghum and maize circumvent the plant’s natural stress response (Zheng et al., 2009; Jordan et al., 2012) and are usually beneficial in an agronomic setting. Reducing other natural stress responses like high water-use efficiency, which often leads plants to leave water in the soil at the expense of potential growth, could be targets of similar innovation (Leakey et al., 2019).

When trade-offs occur within species and there is standing genetic variation in performance or yield, biologists can leverage the possibility of continued evolutionary change to break trade-offs and remove conditionally deleterious variants. In most cases, trade-offs can be broken by consistent selection orthogonal to the direction of the trade-off or by crossing locally adapted genotypes to break up linkage (Conner, 2003; Agrawal et al., 2010) and through multi-trait genomic prediction-based breeding for large mega-environments (Wallace et al., 2018). The trade-offs among locally adapted genotypes that result from conditional neutrality are an ideal target. Since there is no cost of conditionally neutral alleles in a disfavored environment (Anderson et al., 2011), breeding that focuses on combining many conditionally neutral alleles may quickly generate generalist ideotypes, avoiding trade-offs altogether. A similar approach can be used in a molecular context: Kudo et al. (2019) found that drought tolerant rice plants over-express stress-inducible genes, like *DREB1A*, which reduces growth. By breeding plants that co-expressed growth-promoting genes, like *GA5* and *PHYTOCHROME INTERACTING FACTOR4* (*PIF4*), this trade-off could be eliminated (Kudo et al., 2019).

The consequences of failing to break trade-offs can be severe, especially when trade-offs constrain the ability of plant populations to adapt to keep pace with ongoing global change (Chapman et al., 2012). Left alone, species that cannot adapt to rapid global change will need to acclimate or will likely go locally extinct (Hoffmann and Sgrò, 2011; Christmas et al., 2016). In these cases, biologists can also use our understanding of trade-offs to move species with desirable attributes to matching environments (e.g. targeting crop species to the most suitable environments or combining certain species to enhance ecosystem services) (Willi and Van Buskirk, 2022).

Trade-offs are, to some extent, inevitable in biology. But, trade-offs do not need to hamstring our ability to respond to climate change. As the climate becomes more variable and moves further away from historical averages, the target environments for crop breeding will become less obvious (Chapman et al., 2012). Thus, the specialized strategies exhibited by locally adapted crops may become less valuable. A major challenge will be to identify the trade-offs that are most limiting to desired outcomes (e.g. increased production or resilience to climatic extremes) and use our biological insight and engineering principles to break, alleviate, or circumvent those trade-offs. Gene stacking to produce generalist ideotypes and artificial selection in the direction orthogonal to a trade-off are promising approaches that may be feasible in the short term. In the longer term, more extreme interventions, like genetically engineering crops to use C4 photosynthesis or to become perennials, may be required to break or alleviate some of the most recalcitrant trade-offs. Given the importance of trade-offs for so many aspects of biology, their study can be a promising approach for enhancing the resilience of our agricultural and natural systems in an increasingly variable world.

## How does the circadian clock “gate” plant responses to abiotic stress?

### (By Paloma Mas)

Severe drought, extreme temperatures, and changes in salinity all disturb plant cellular homeostasis and cause deleterious effects on crop growth and productivity. Throughout evolution, plants have developed a battery of responses to reach a cellular status that is tolerant or compatible with harsh conditions (Markham and Greenham, 2021). Understanding the array of responses triggered by abiotic stress can provide useful information to obtain crop varieties adapted to stress (Iannacone et al., 2012; González-Guzmán et al., 2022). Among the different signaling pathways involved in plant responses to stress, the circadian clock stands out as a main cellular mechanism able to measure time and to coordinate key biological processes in synchrony with the environment (Sanchez and Kay, 2016). The proper function of the circadian clock enables plants to anticipate the daily changes in the environment, controlling the timing of growth, development, and responses to biotic and abiotic stresses (Sanchez and Kay, 2016).

Mutation of clock components disturbs the ability of plants to adapt to environmental stress conditions (Seo and Mas, 2015; Bonnot et al., 2021). The number of stresses connected with the circadian clock is ample and includes drought, heat, cold, or redox imbalance (Grundy et al., 2015). Interestingly, the circadian clock not only regulates daily or seasonal oscillatory stresses such as heat stress during the day or severe cold during the night, but also continuous stresses with no obvious oscillations, like high salinity (Park et al., 2016). The anticipation provided by the circadian clock may enable plants to prepare in advance against stresses that diurnally or seasonally oscillate, and thus conferring an adaptive advantage. However, the regulation of constant stresses may rely on specific clock components that act independently of their function within the clock. It is also possible that the constant stress is related to other stresses and pathways in which circadian timing is relevant.

Based on the pervasive role of the circadian clock in the regulation of abiotic stress responses, the focus of attention is directed toward the use of the circadian system for improved tolerance to a broad combination of stresses without imposing detrimental pleiotropic effects such as growth arrest, or reduced yield. To that end, multiple strategies can be adopted, but particularly interesting are those related to a central function of the circadian system, known as “gating,” whereby the clock differentially regulates the magnitude of the plant response to environmental signals depending on the time-of-day (Seo and Mas, 2015). The circadian peak of expression of many genes involved in stress responses coincides with the recurrent peak time of the stress (Bonnot et al., 2021), and so, a large proportion of the heat- and cold-responsive transcriptome is gated by the clock (Covington et al., 2008; Blair et al., 2019). Interestingly, the clock is also able to gate stress responses depending on the time of the year (Lee and Thomashow, 2012). For example, key factors involved in cold responses oscillate with higher amplitude under short-day conditions, providing improved tolerance to cold conditions during the winter time. Thus, using circadian gating might be advantageous for plants to restrict their response only to the most appropriate and needed time, as opposed to a constitutive response normally associated with high energy demand and with the penalty on growth and yield.

**Figure 6:**
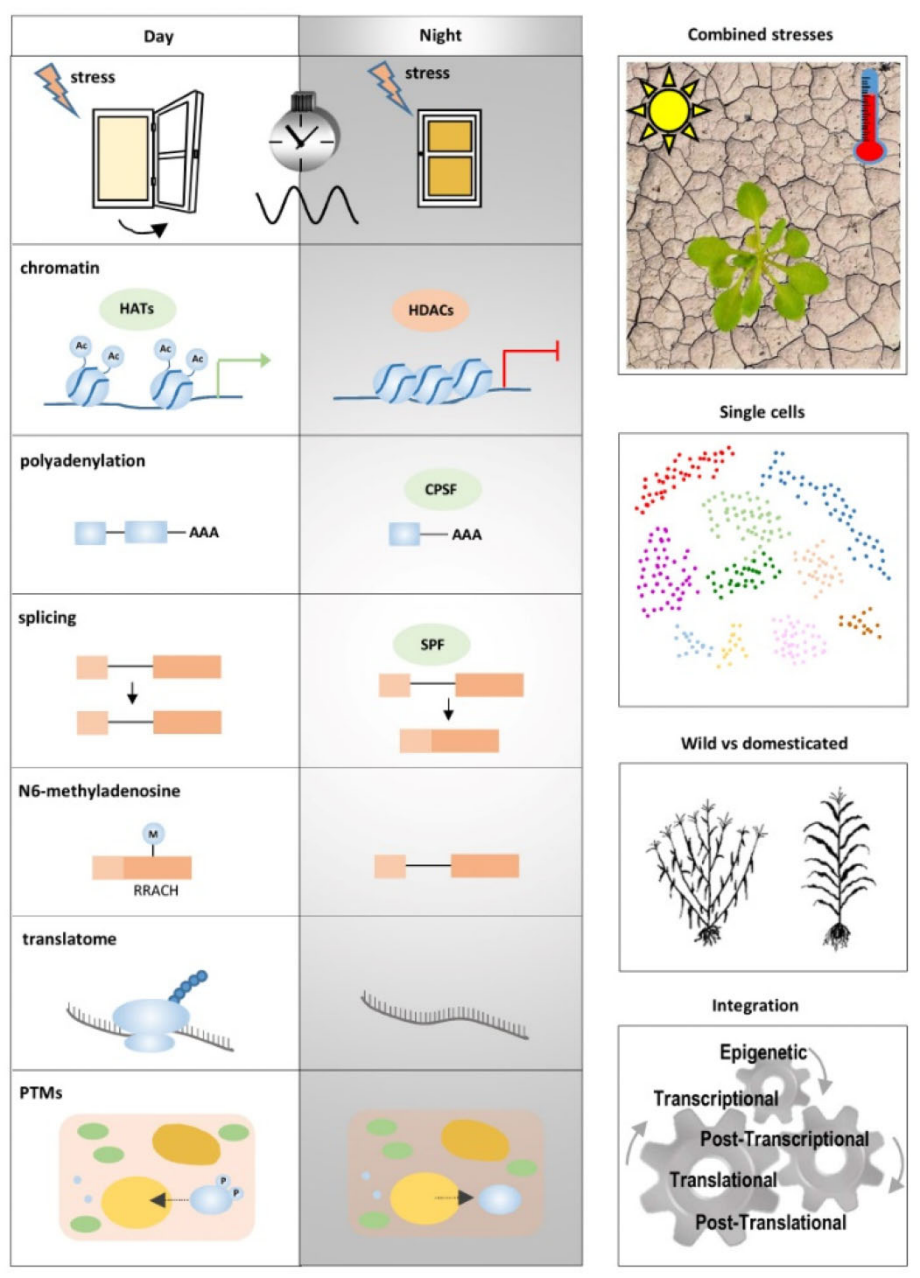
Circadian gating by the clock differentially regulates plant responses to stress depending on the time of day. Figurative examples of open questions related to the circadian gating via changes in chromatin, posttranscriptional, translational, and posttranslational mechanisms of regulation that ultimately define gene expression and protein function under stress conditions. Single-cell approaches with plants grown under conditions that closely follow the natural environment, including combined stresses, and comparisons of domesticated crops and their wild relatives are circadian research areas of increasing interest. Understanding the coupling among the different gating mechanisms will pave new ways for using the circadian clock to tackle the effects of abiotic stress on plants.

But, what are the mechanisms behind the gating function and regulation? Although most studies have focused on transcriptional control, posttranscriptional regulation can be also gated by the clock. For example, alternative splicing has been associated with heat stress responses (Ling et al., 2021), and Splicing Factor 30 could be one of the many plausible candidates linking the clock with alternative splicing and heat stress (Bonnot and Nagel, 2021). Identifying all the components of the splicing machinery gated by the clock, and the alternative splicing isoforms functionally relevant under single or combined stress conditions is an interesting area of ongoing and future research. Similarly, studies showing the connection of alternative polyadenylation with the clock (Yang et al., 2020), and with stress (Yang et al., 2021) open the way for functional studies on the circadian gating of alternative polyadenylation. New areas of study can specifically focus on the circadian coupling of transcription with polyadenylation, splicing, or RNA modification (N6-methyladenosine, 5-methylcytosine, or pseudouridine) (Yang et al., 2021). The clock also selectively redefines the pool of mRNAs to be translated under heat stress, controlling about one-third of the circadian- and heat-dependent translated proteins (Bonnot and Nagel, 2021). Expanding these studies to other abiotic stresses will provide a global view of how circadian clock gating impacts the abiotic stress-related translatome. The key role of posttranslational modifications on abiotic stress responses (Hashiguchi and Komatsu, 2016) also paves the way for future studies on how circadian gating controls other posttranslational modifications such as phosphorylation, ubiquitination, glycosylation, etc. to regulate protein function and localization in response to abiotic stress (Figure 6).

Changes in DNA methylation and histone modifications result in epigenetic variation that provides phenotypic plasticity and plant adaptation to changing environments (Miryeganeh, 2021). As the circadian clock is also closely connected with chromatin remodeling (Chen and Mas, 2019), it would be interesting to fully explore the circadian gating of epigenetic responses to stress. Research could focus on identifying the full array of chromatin “readers” and “erasers” controlled by the clock and their connection with the stress-responsive loci. The fact that epigenetic marks can trigger stress memory in primed plants also places the spotlight on identifying the role of the clock in providing a long-term memory for the stress response. To obtain meaningful results, the stress-related experiments should be performed under growing conditions that mimic as close as possible the natural growth environment (Panter et al., 2019). Likewise, analyzing combinatorial stresses that usually appear simultaneously or sequentially in nature (e.g. heat and drought) might provide much more reliable and relevant information to use for enhancing crop tolerance to abiotic stress (Rivero et al., 2022). Studies could also switch the focus from whole plants to single cells in order to get meaningful conclusions about stress perception, signaling, and responses in specific cell types. Also, looking back to plant ancestors and comparisons of domesticated crops and their wild relatives (Markham and Greenham, 2021; Figure 6) will surely provide novel avenues to understand how the circadian gating by the clock can be exploited for adaptation to environmental stresses, thus providing novel opportunities for targeted approaches for improved tolerance to abiotic stress (Bhatnagar-Mathur et al., 2008).

## How do lipid-derived second messengers translate abiotic stress information into cellular responses for stress acclimation?

### (By Teun Munnik)

Phospholipids are crucial building blocks for membrane function. They create the bilayered, liquid structure that surrounds every cell, organelle, and endosomal compartment, and hosts thousands of integral and peripheral membrane proteins essential for membrane energization, signal detection and transduction, as well as primary and secondary metabolism (Munnik et al., 2021). Phospholipids are indispensable for cell function (Noack and Jaillais, 2020).

Besides a structural function, phospholipids have also emerged as crucial signaling molecules, either as precursors of signaling molecules or as lipid second messengers themselves. The best examples come from polyphosphoinositides (PPIs), which are inositol-containing phospholipids that can be phosphorylated at the D-3, D-4, and/or D-5 position of the inositol ring, thus creating five distinct molecular species in plants (i.e. PI3P, PI4P, PI5P, PI(3,5)P_2_, and PI(4,5)P_2_) as well as two more that are present in metazoans but have not been detected in plants [PI(3,4)P_2_ and PI(3,4,5)P_3_] (Munnik and Testerink, 2009; Gerth et al., 2017). PPIs are typically low abundance lipids that escape detection by common mass spectrometry methods, and turn over rapidly. They act as biochemical and biophysical landmarks that contribute to membrane identity, signaling, and compartment morphodynamics, with each PPI species accumulating in different set of endomembranes thus helping to define membrane identity (Gerth et al., 2017; Dubois and Jaillais, 2021).

In the world of PPI signaling in animals there has been something of a revolution over the last decade that has manifested itself in an increasing appreciation of PI(4,5)P_2_ as a regulator of cellular events in its own right. PI(4,5)P_2_ has been known for a long time to fulfill a crucial role as the substrate for two major signaling pathways: the phosphoinositide (PI)–phospholipase C (PLC) pathway that generates inositol-1,4,5-trisphosphate (IP_3_) and Ca^2+^ release, as well as diacylglycerol (DAG) to activate protein kinase C (PKC); and the PI 3-kinase pathway that generates PI(3,4,5)P_3_, a crucial lipid second messenger in regulating cell proliferation and many other physiologically important processes (Balla, 2013; Dickson and Hille, 2019).

Yet, higher plants clearly have a different agenda. They do have the PI–PLC pathway to generate the above-mentioned signaling molecules, but they have no recognizable PKC or IP_3_ receptors, and no PI 3-kinases of Type I, the group that uses PI(4,5)P_2_ as a substrate (Munnik and Testerink, 2009). Yet Arabidopsis has 11 Type I PIP 5-kinases (PIP5Ks), nearly four times as many as us animals! And why despite this do plants maintain PI(4,5)P_2_ levels 1–2 orders of magnitude lower than metazoans? The only logical answer to these questions is that PI(4,5)P_2_ has an even more central role as a signaling entity in its own right than it does in animal biology. In plants, there seems to be a proliferation of PI(4,5)P_2_ functions in conjunction with an increasing understanding of PI(4,5)P_2_ compartmentalization (Munnik, 2014; Doumane et al., 2022). Together, these new insights are revolutionizing how we think about this ancient and original PPI.

Knockout of some Arabidopsis PIP5Ks (PIP5K1–PIP5K6) clearly indicate a role for PI(4,5)P_2_ in cell polarity, in particular during cell division and polar growth of root hairs and pollen tubes (van Leeuwen et al., 2007; Kusano et al., 2008; Ischebeck et al., 2010; Tejos et al., 2014). In contrast, no or only subtle developmental phenotypes are observed for PIP5K7–PIP5K9 mutants as these PIP5Ks are instead involved in salinity stress and response to polyamines (Zarza et al., 2020; Kuroda et al., 2021). Genetically encoded-PI(4,5)P_2_ biosensors (van Leeuwen et al., 2007; Simon et al., 2014) revealed that PI(4,5)P_2_ typically accumulates at the plasma membrane, except during heat stress where additional punctates appeared in cytosol and near the nuclear envelope (Mishkind et al., 2009). What these punctate compartments are, and which PIP5K generates them, is still unknown. Genetic manipulation of PI(4,5)P_2_ by inducible production or depletion revealed crucial roles for PI(4,5)P_2_ in endocytosis and regulating the actin- and microtubule cytoskeleton, with dramatic consequences for development (Gujas et al., 2017; Doumane et al., 2021).

But how is PI(4,5)P_2_ managing all this? In metazoan systems, several protein targets have been characterized, including PI(4,5)P_2_ specific-binding domains, for example, PH, Tubby, and SEC14 (De Jong and Munnik, 2021). While several plant proteins contain such domains, their PI(4,5)P_2_-binding specificity and functionality has remained largely unexplored (De Jong and Munnik, 2021). Since plant cells contain much lower PI(4,5)P_2_ levels than animals, and since it is no problem to stably express PI(4,5)P_2_-biosensors (based on exogenous PH and Tubby domains) in Arabidopsis without causing any phenotype (van Leeuwen et al., 2007; Simon et al., 2014), plants are likely to contain distinct PI(4,5)P_2_-binding domains with a much higher affinity. Such domains, however, remain to be identified. In animals, the gating of most K^+^ channels is regulated by PI(4,5)P_2_, and there are indications this occurs in plants too (Zarza et al., 2020).

Phosphatidic acid (PA) is another important plant lipid second messenger that is typically triggered upon abiotic stress, including heat, cold, drought, and salinity stress, but also in response to pathogens and wounding (Munnik, 2001; Kim and Wang, 2020). In general, PA responses are fast (min) and generated through hydrolysis of structural phospholipids by phospholipase D (PLD) and/or through phosphorylation of DAG by DAG kinase (DGK) (Munnik and Testerink, 2009). Arabidopsis contains 12 PLDs and 7 DGKs, and 9 PI–PLCs that produce DAG by hydrolyzing PI4P or, if generated, PI(4,5)P_2_. How PA is involved in the different stress responses, via which PLD, DGK, and PLC, and at which cell or compartment this takes place, has been a central theme of the last decade, and will still be in the next. Complementary approaches include KO and OE mutants on PA production, isolation, and characterization of PA targets, and the construction of genetically encoded-PA biosensors to monitor PA in living cells (Munnik and Testerink, 2009; Platre et al., 2018; Kim and Wang, 2020; Scholz et al., 2022). While these tools helped to establish PA as a lipid second messenger that rivals the importance of Ca^2+^, we still know very little of either PA versus Ca^2+^ specificity in responding to different stresses or their potential collaboration. Monitoring both molecules simultaneously with ratiometric biosensors would certainly help clarifying this.

**Figure 7:**
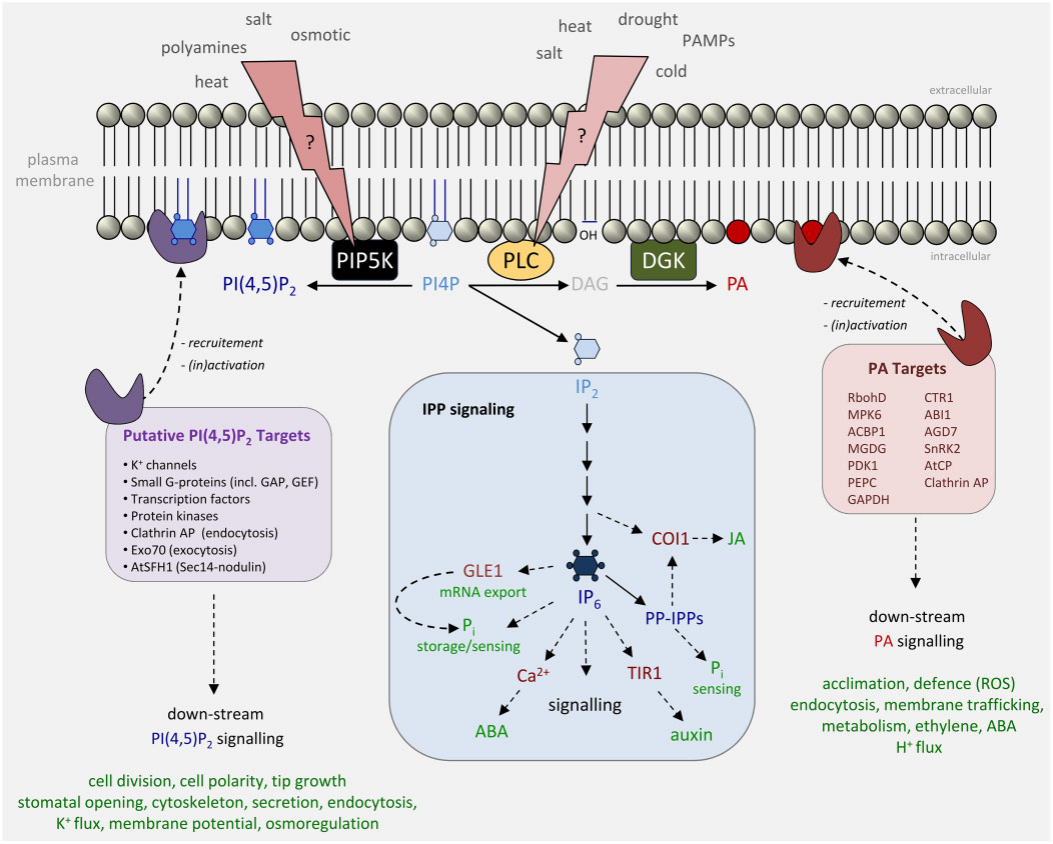
Possible signaling mechanisms of PI(4,5)P_2_, PA, and PLC in plant abiotic-stress signaling. PI(4,5)P_2_ concentrations in plants are extremely low but synthesis is rapidly triggered by activation of one or more of the 11 PIP5Ks of Arabidopsis via unknown receptors or activation mechanisms (indicated by “?”). As such, PI(4,5)P_2_ is triggered by heat, salinity, osmotic stress, and polyamines, but also during polar growth, predominantly accumulating at the plasma membrane. This likely causes recruitment of target proteins to the membrane or (in)activation of targets that are already present at the membrane. Putative plant targets are based on the mammalian literature and correlations with plant literature. Plant PLCs likely hydrolyse PI4P and generate membrane-bound DAG and the water-soluble headgroup, IP_2_. Again, receptors and activation mechanisms remain unknown (indicated by “?”). The newly formed DAG is rapidly phosphorylated by DGK to form the signaling lipid PA for which several protein targets have been identified. IP_2_ can be stepwise phosphorylated by IPP multi-kinases to produce IP_5_ and IP_6_, and IPP-pyrophosphates IP_7_ and IP_8_, for which several signaling functions and targets are emerging. Protein targets are indicated in purple [PI(4,5)P_2_] or dark red [PA]. Lipid (-derived) signals are indicated in blue. Solid lines indicate metabolic conversions; dashed lines represent “activation” or “cause.” Effects are indicated in green.

Another challenge remaining is to understand PI–PLC signaling in plants (D’Ambrosio et al., 2017; van Wijk et al., 2018; Zhang et al., 2018c, 2018d). In vitro, the enzyme equally likes PI4P and PI(4,5)P_2_ as substrate, but the general absence of PI(4,5)P_2_ in plant plasma membranes, while PI4P is relatively abundant, makes it more likely that IP_2_ is generated rather than IP_3_ in vivo. This would also explain the lack of IP_3_ receptors (Munnik and Vermeer, 2010; Munnik, 2014). For DAG, it makes no difference to be converted into PA. IP_2_ can be step-wise phosphorylated into various inositolpolyphosphates (IPPs) that are emerging as signaling molecules, including IP_5_ and IP_6_, but also IPP-pyrophosphates IP_7_ and IP_8_ (Lorenzo-Orts et al., 2020). IP-related signaling functions in plants include intracellular Ca^2+^ release (ABA), acting as co-factor for auxin and jasmonate signaling (via TIR1 and COI1), as well as functions in RNA transport and P_i_ sensing (Figure 7; Munnik, 2014; Lorenzo-Orts et al., 2020). Linking all these cellular phenotypes to PI–PLC signaling remains challenging (Zhang et al., 2018c, 2018d).

While the above mostly deals with questions downstream of abiotic stress-triggered lipid signaling, we are equally in the dark on how lipid kinases, phosphatases, and phospholipases are actually activated upstream. The next decade will be an exciting venture into a much deeper understanding of how plants use signaling lipids and inositol phosphates to control their physiology and respond to stress. It will not just widen our basic knowledge of plant biology, but very likely also our understanding of stress responses that will most benefit efforts to improve plant resilience.

## How can laboratory stress research be applied to continuously stressed plants in the field?

### (By Hilde Nelissen)

One of the outstanding questions in plant abiotic stress biology is why the vast amount of knowledge on physiological and molecular responses to abiotic stresses has resulted in so few climate resilient crop varieties that are currently on the market (Sanchez, 2013; Blum, 2014; Nuccio et al., 2018). Among the possible reasons that hamper the translation of molecular knowledge toward application, the gap between environmentally controlled conditions versus more variable field conditions was identified as one of the bottlenecks (Nuccio et al., 2018). Controlled conditions, here used as a collective name for greenhouse and growth chamber experiments, allow higher throughput, increased speed of innovation and reduced cost compared with field trials (Simmons et al., 2021). In addition, more detailed mechanistic insights and mode of action studies often require controlled conditions, where responses to changes in the environment can be monitored with respect to time, space, amplitude, and other factors related to both the stress applied and the response output (Verslues et al., 2006; Bao et al., 2014; Alejo-Jacuinde et al., 2022; Hu et al., 2022). On the other hand, field trials provide valuable information about the potential of a stress-tolerant product for marketable yield stability without yield drag. However, field evaluations are costly and labor-intensive because of the experimental design and replication needed to detect a desired effect, management logistics, and weather hazards. Thus, given the throughput and the costs involved, the logic pipeline for plant improvement is a funnel-like screening in which many lines are screened in controlled conditions, of which the most promising leads subsequently undergo field evaluations (Simmons et al., 2021). However, the realization is growing that the inability of typical laboratory- or greenhouse-controlled conditions to properly model agronomic environments hampers the translation of knowledge on abiotic stresses to climate resilient crops (Sanchez, 2013; Blum, 2014; Nuccio et al., 2018; Simmons et al., 2021).

As the name implies, controlled conditions aim to maximally standardize the growing conditions to reassure reproducibility and to facilitate detailed studies on the effects of a limited perturbation. In controlled condition stress experiments, typically temperature, humidity, irrigation, or light is altered or a substance is added to mimic an abiotic stress response. Ideally, the other environmental parameters are kept as standardized and stable as possible, so that only one or a combination of a few abiotic stresses occur simultaneously. However, to fully understand the impact of the applied stress(es), it might be necessary to monitor additional parameters, such as soil water content in drought experiments. The stress(es) can be maintained for a substantial time of the plant’s development, sometimes followed by stress alleviation, but more frequently stress treatments in greenhouse and growth chamber conditions are short and severe to evoke molecular changes. The setup of such an “ideal” laboratory stress assay is often more dependent on the greenhouse and growth chamber facilities and the assay robustness than on reflecting the actual field conditions.

Anyone who has performed field trials or has a private garden realizes that there is no typical growth season that could be represented by standardized, control conditions. Even when temperature and precipitation are close to the multi-year average, there will be periods of extreme weather conditions. Besides the conspicuous extreme weather conditions, there are also less obvious consequences of climate change that can have detrimental effects on crop yield. For example, the global rising temperature is not only experienced during the daytime. Increased night temperature is also a problem for plants in that it enhances night respiration that increases utilization of photo-assimilates and thereby reduces the amount of carbon available for grain filling (Desai et al., 2021; Xu et al., 2021). In the field, some stresses are continuously present, like heavy metals in the soil, but other stresses build up more gradually, like drought, and can occur during any developmental stage with distinct impacts on yield (Verbraeken et al., 2021). In field conditions, stresses rarely occur alone or in a coordinated way, and temperature, humidity, irrigation, light, and several additional factors fluctuate continuously rendering the plants constantly stressed.

The fact that field-grown plants are continuously in a stressed state is reflected by the transcriptomic changes between plants of the same genotypes, grown in controlled conditions and multiyear field trials. Several stress-related genes that confer abiotic stress tolerance in over-expression lines exposed to controlled stress conditions were massively upregulated in field-grown versus laboratory-grown plants, even under relatively normal field conditions (Nelissen et al., 2020). Even neighboring plants of the same genotype grown in one field displayed transcriptional differences in stress-related genes (Cruz et al., 2020). Studies that also take into account the weather conditions when analyzing field transcriptomics (Nagano et al., 2012) or that incorporate measurements of plant water status show that individual field-grown plants sense a micro-environment and react molecularly to local changes in environmental changes by altering stress-related genes, some of which were already identified in controlled conditions.

We showed that not only known stress-related genes were differentially expressed between the controlled conditions and the field (Nelissen et al., 2020). Genes involved in processes such as shade avoidance were also differentially expressed between the laboratory and the field, which, in turn, may interact with stress responses (Hayes et al., 2019), complicating the situation even more. In addition, shade avoidance is a response that breeding attenuated to achieve higher planting density, an agronomic practice that is often overlooked in laboratory or greenhouse experiments. In such experiments, plants are grown in pots filled with potting soil, so that individual plant irrigation schemes or nutrient applications can be monitored. The pots are arranged to optimize greenhouse space, irrespective of whether the plants under study are typically grown as high-density row crops or subjected to other management regimes. In this way, differences in inter-plant population dynamics, interactions with other organisms, or effects of crop rotation or intercropping are not accounted for when screening in controlled abiotic stress conditions. By individualizing every plant, field-born local differences that cause molecular responses (Cruz et al., 2020) or trigger differences in developmental programming (Robbins and Dinneny, 2015) can be overlooked, along with the effects of soil differences related to recent cropping history or biotic interactions (Beirinckx et al., 2020).

**Figure 8:**
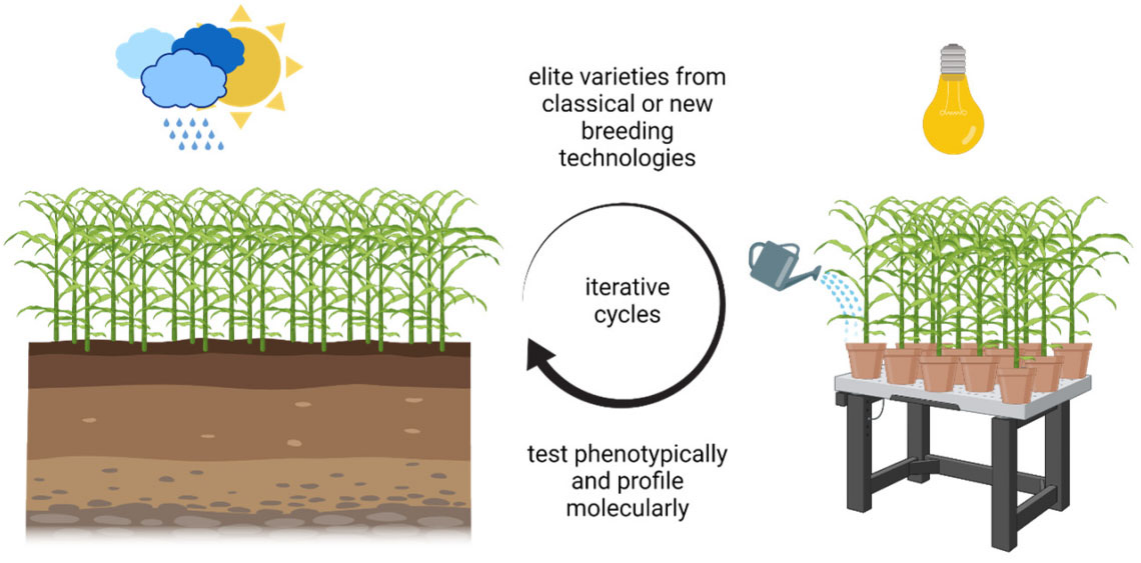
Schematic representation of the differences in management, light, and irrigation schemes between plants grown in the field (left) and in controlled conditions. Iterative cycles of field testing and molecular profiling of all lines with putatively improved traits will be necessary to increase our understanding how the stresses that plants experience in the field relate to the controlled abiotic stress conditions. Created with BioRender.com.

The fact that abiotic stress-related genes, identified in controlled abiotic stress experiments, are transcriptionally regulated in the field, confirms their potential usefulness in improving plant resilience. However, this also urges plant scientists to gain a better insight into the molecular “state of mind” of field-grown plants in order to come up with more clever ways to modulate these genes and the corresponding networks to improve plant performance. To achieve this, the fields of breeding and molecular biology need to work together more closely to grow and molecularly profile new varieties with improved traits in both field and controlled conditions, irrespective of whether the lines were generated by traditional breeding, genome editing, or transgenic approaches. This would enable a virtuous cycle of each new variety to be field phenotyped and molecularly profiled, providing information for further improvements (Figure 8). However, for such efforts to have a maximal impact on agriculture, there is also a need to ease and harmonize regulations that govern the use of genome-edited or transgenic crops for sustainable agriculture and food production.

## Where is the plant most hydraulically vulnerable?

### (By Lawren Sack, Craig Brodersen, and Thomas N. Buckley)

When storms threaten the power grids distributing electricity across a continent, we need to know their weakest links to better prepare for and quickly remediate calamitous failures (Weiss and Weiss, 2019). Equally, we need to ask, as increasingly frequent droughts face plants throughout our globally important and vulnerable ecosystems (Hammond et al., 2022), where are their weakest points? That is, where within the plant does the impact of water stress trigger the most severe reductions in leaf gas exchange and whole plant productivity, the most irrecoverable damage, and the greatest risk of mortality—especially with aggravating stresses such as insect outbreaks and fire. Addressing this question is critical to predicting the impacts of climate change on the local and global distribution of ecosystems, the future of agricultural and forest gas fluxes, and even the behavior of the atmosphere and climate system. Equally, answering this question will inform the breeding of drought-resistant crops for food security.

**Figure 9:**
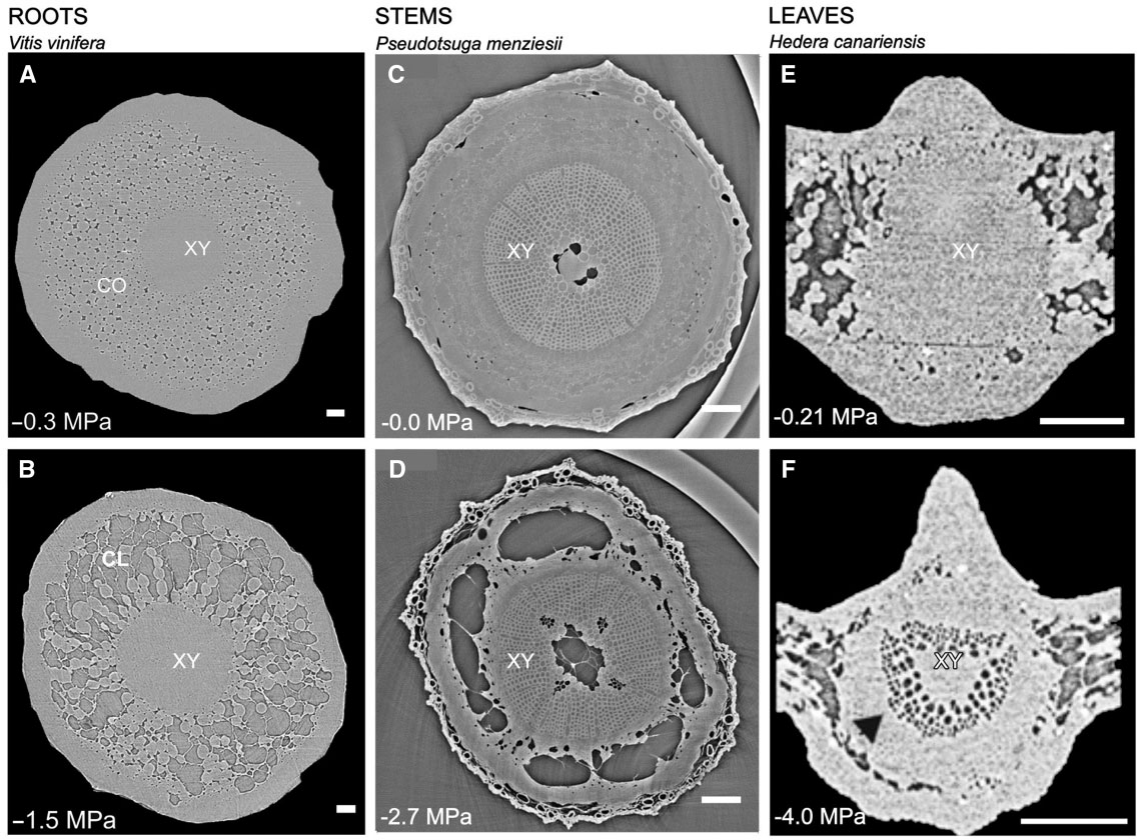
The effects of drought on tissues across plant organs. Representative transverse X-ray micro-computed tomography images show the in vivo functional status of the vascular system and the surrounding tissues under well-watered (A, C, E) and drought (B, D, F) conditions in roots, stems, and leaves. In (A) and (B), the roots of grapevine (*Vitis vinifera*) the xylem network (XY) remains functional under a range of water potentials (*ψ*_w_) but mild drought (−1.5 MPa) leads to massive cellular damage and formation of cortical lacunae (CL in B) that physically decouple the vascular system from the surrounding soil (Cuneo et al., 2021). In (C) and (D), the vascular systems of the immature stems of conifer seedlings (*Pseudotsuga menziesii*) are highly drought tolerant (XY), but deformation of the cortex under strong drought in these resembles that in roots (Miller et al., 2020). In (E) and (F), leaves of ivy (*Hedera canariensis*), subjected to very strong dehydration (−4.0 MPa) show xylem cavitation in the midrib (XY) and the strong mesophyll tissue deformation that occurs during drought exceeding the turgor loss point (Scoffoni et al., 2017). Bars = 200 µm in (A) and (B); 100 µm in (C) and (D); 250 µm in (E) and (F). Values in the lower left corner of each panel indicate the *ψ*_w_ of the plant during the experiment.

Sensational or not, the analogy of plants as power grids is in fact a well-established quantitative approach. Much understanding of plant hydraulics arose from the classical comparison of the soil–plant–atmosphere continuum to an electrical circuit, where flow paths are resistors and water potential gradients are voltages, subject to the application of the analogy to Ohm’s Law (Van den Honert, 1948; Tyree and Zimmermann, 2002):

ΔV=IR,

where *V* is the voltage, *I* the current, and *R* the electrical resistance,

By analogy,

$$\Delta \psi_{w}=ER,$$

where *ψ*_w_ is the water potential, *E* the transpiration rate, and *R* the hydraulic resistance. Thus, the stronger the flow rate, and the resistance to water movement, the stronger the drop in *ψ*_w_ (or pressure) across the organ, or whole plant, between the soil and the atmosphere; conversely, the stronger the *ψ*_w_ gradient driving force, the stronger the flow rate through the system or any component. This electrical analogy stands in for more detailed theory of fluid mechanics and irreversible thermodynamics (Edlefsen, 1941; Slatyer and Taylor, 1960; Gibbs, 1961; Granger, 1995), and can have drawbacks (namely, when factors other than *ψ*_w_, such as thermal gradients drive water movement; Rockwell et al., 2014; Buckley et al., 2015). On the other hand, the analysis of the plant and its environment as a network of electronic components yields a wealth of predictions and mechanistic representations by considering analogies of tissues, plants, and ecosystems as including fixed resistors, variable resistors (potentiometers), capacitors, and diodes. Studies using this approach to analyze the distribution of hydraulic resistance within the plant network suggest that the extremities of the plant represent key bottlenecks to water flow, with the leaves and roots accounting for >75% of total resistance and stems <25%, in a wide variety of growth forms (Tsuda and Tyree, 2000; Sack et al., 2003; Domec et al., 2009). Moreover, these resistances are dynamic, subject to internal and environmental control. Most famously, in every component of the system, hydraulic conductance (*K*), the inverse of resistance, declines precipitously at lower *ψ*_w_, a “hydraulic vulnerability” arising from a multiplicity of processes (Figure 9, A–F). In soils, air replaces water and thus removes flow paths for water to be sucked into the plant. In xylem throughout the plant, under strong dehydration, cavitation occurs, which is the formation of air- or vapor-filled conduits that block water flow (Figure 9, E and F; Tyree and Zimmermann, 2002), However, in leaves and roots, water flows not only through xylem but also through living tissues (on the way from the soil to the root xylem, and from the leaf xylem to the stomata). Before embolism forms in the xylem, in dehydrating roots, cortical lacunae may form that break the hydraulic connection between the root xylem and the soil (Figure 9, A and B; Cuneo et al., 2021), and in dehydrating stems and leaves, tissues shrink, which may influence flow pathways around and within cells, as the water channel proteins that traverse cell membranes, known as aquaporins (AQPs), can be gated even under mild dehydration (Scoffoni et al., 2014; Figure 9, C–F). Given their extra-xylem pathways, and despite similarity of their xylem in resistance to embolism (Zhu et al., 2016a, 2016b; Klepsch et al., 2018; Li et al., 2020; Smith-Martin et al., 2020), leaves and roots tend to be more hydraulically vulnerable than stems (Scoffoni et al., 2017; Albuquerque et al., 2020; Cuneo et al., 2021).

Does the combination of the greater bottlenecks and vulnerabilities within leaves and roots than stems make these extremities the plant’s most sensitive fuses under strong drought? Indeed, many have concluded that these organs should be more vulnerable, to protect stem xylem from tensions that would drive irreparable cavitation, as the stem is longer-lived and more costly to replace, ideas known as the “hydraulic-segmentation” and “vulnerability-segmentation” hypotheses (Tsuda and Tyree, 1997; Tyree and Zimmermann, 2002; Pivovaroff et al., 2014). Yet, the challenge faced by plant organs will shift as drought proceeds, along with the distribution of different *ψ*_w_ within the plant. In drought mild enough that stomata remain open, transpiration causes *ψ*_w_ to be lowest in the leaves, and given that the leaf is a bottleneck and highly vulnerable, *K* loss in leaves can be drastic (Hernandez-Santana et al., 2016; Scoffoni and Sack, 2017; Albuquerque et al., 2020). Leaf *K* loss may, however, act as a brake on water loss by amplifying stomatal closure (Scoffoni and Sack, 2017). In severe droughts, where turgor is lost and stomata are fully closed, the plant will be close to equilibrium with the soil, with all organs experiencing similar *ψ*_w_. As the soil and plant dehydrate further, exacerbated by the low “minimum conductance” from incompletely closed stomata and/or across the cuticle (Martin-StPaul et al., 2017), the leaf xylem may suffer embolism, starting with major veins and proceeding to minor veins, and this embolism may trigger leaf death (Brodribb et al., 2021). When substantial cavitation occurs in the stem xylem, it usually kills the stem, because cavitation tends to beget more cavitation, in a vicious cycle of “catastrophic embolism”—with gas bubbles spreading, uncontrolled, among conduits (Tyree and Zimmermann, 2002). As for the roots, the vulnerability of their xylem and outside-xylem pathways may also be strong (Brunner et al., 2015; Rodriguez-Dominguez and Brodribb, 2020). Across species, this sequence of hydraulic decline—leaves before stems and roots—tends to be typical, and species’ thresholds for declines of *K* in organs and the effects of drought-induced damage are correlated (Bartlett et al., 2016; Dayer et al., 2020).

This overall simple scenario, however, has been tested in a few hundred plant species at best, and not in sufficient detail to fully parameterize the hydraulic network for the bulk of plants. Particularly little is known of the hydraulic vulnerability of roots, due to technical difficulties; recent studies on potted plants of a few species have proposed that the decline of hydraulic conductance in the root and/or root–soil interface can be strong enough even in relatively moist soil to contribute to stomatal closure (Abdalla et al., 2021; Bourbia et al., 2021; Duddek et al., 2022). Further, the potential role of the root sheath and mycorrhizae in modulating or perhaps protecting root and root interface hydraulic conductance has yet to be fully clarified (e.g. Boomsma and Vyn, 2008; Brunner et al., 2015). Indeed, the fine roots are more vulnerable than older roots, and their vulnerability needs separate quantification, especially if fine root death and turnover occur even under mild soil moisture deficit (Cuneo et al., 2021).

Given these numerous unknowns, the location of hydraulic triggers for declines in gas exchange and death is a critical avenue for research. When enough hydraulic conductance is lost, water cannot be transported into and throughout the plant, and a spiral of mortality begins, potentially exacerbated by carbon starvation and other biotic and abiotic stresses (Choat et al., 2018; Hammond et al., 2022; McDowell et al., 2022). Yet, water storage can buffer given organs from loss of hydraulic conductance, and protect the plant from dehydration, especially when the plant retracts its roots to prevent water loss to the soil. The general magnitude and role of water storage “capacitors” is another, related critical unanswered question (McCulloh et al., 2014; Knipfer et al., 2019). Least of all is known about the triggers for death, and their timing and general order during dehydration, for cells within tissues, tissues within organs, and organs throughout the plant. Indeed, new concepts are needed—there is no generally agreed definition for the time of death of an organ, tissue, or whole plant—and plants may be incredibly diverse in this death pattern (Hammond and Adams, 2019). In many species, leaf cells are apparently damaged or killed by dehydration below the turgor loss point, but in resurrection plants, cells can recover completely (Stuart, 1968; Alpert, 2000; Prats and Brodersen, 2021). In deciduous species, the leaves die first, and then the buds on the stem, but in some species, roots apparently die early on and spell the death of the plant (Sack, 2004). Plants with multiple stems and/or sectoriality among stems and roots may better survive drought due to redundancy and/or resprouting after mortality of stems and roots (Schenk et al., 2008; Zeppel et al., 2015; McElrone et al., 2021). Answering the critical question of the location of hydraulic bottlenecks, vulnerabilities, and triggers of mortality within plants, and of the traits with predominant influence across diverse plants, will open the door to the prediction of plant mortality and ecosystem shifts, and the design of drought-hardy crop varieties. These imperatives are as urgent as readying our power grids for the storms of climate change.

## How does the continuing rise in CO_2_ affect the regulation of stomatal apertures and water-use efficiency of plants?

### (By Julian I. Schroeder and Po-Kai Hsu)

The atmospheric CO_2_ concentration is continuing to rise and is now ∼50% larger than before the industrial revolution. This is resulting in increased absorption of infrared radiation by CO_2_, which in turn is causing temperatures to rise on Earth. Plants remove CO_2_ from the atmosphere via photosynthesis. CO_2_ can be viewed as an abundant atmospheric fertilizer that contributes to plant growth, if nutrients and water are sufficiently available and depending on the species and conditions (De Kauwe et al., 2021). Indeed, satellite monitoring of photosynthesis and plant growth has shown global “greening” (Zhu et al., 2016a, 2016b; Chen et al., 2022). However, warming temperatures globally can increase vapor pressure deficit, which arguably may tend to counteract this greening trend (Zhu et al., 2016a, 2016b; Wang et al., 2020a, 2020b), with this hypothesis being a matter of debate and further studies being warranted.

CO_2_ enters leaves for photosynthesis via stomatal pores. Typically, plants lose ∼150 to >500 water molecules via stomatal transpiration for every CO_2_ molecule that is taken in and assimilated by photosynthesis. The CO_2_ concentration itself is a regulator of the rapid stomatal closing and opening responses. Elevated CO_2_ in the intercellular spaces of leaves, [CO_2_]*i*, occurs at night in C3 and C4 plants due to respiration, triggering stomatal closing. During light periods, photosynthesis reduces [CO_2_]*i*, which mediates stomatal opening, together with a light-triggered signal transduction network. The atmospheric [CO_2_] rise is adding to these diurnal changes in [CO_2_]*i*, thereby causing a narrowing of stomatal pores globally (Medlyn et al., 2001; Franks et al., 2013). This CO_2_ response can be beneficial to plants. Reduction in stomatal apertures resulting from elevated atmospheric [CO_2_] can enable plants to maintain photosynthetic CO_2_ assimilation rates, while losing substantially less water, thereby improving water use efficiency (WUE). However, there are limitations, including that many plants have a “weak” stomatal CO_2_ response (Raschke, 1975), thereby showing less or no improvement in WUE. C_4_ plants show saturation of assimilation at relatively low [CO_2_]*i* levels and reduction of stomatal conductance may improve WUE depending on the species and conditions (De Kauwe et al., 2021). Moreover, this is also relevant for C_3_ plants (Doheny-Adams et al., 2012), including in forestry, where reduction in transpiration from trees could slow depletion of soil water content.

On the other hand, in agricultural regions with sufficient rainfall, soil nutrients, and favorable growth conditions, the high CO_2_-induced reduction in stomatal apertures could limit photosynthesis. This applies particularly to C3 plants for which photosynthetic CO_2_ assimilation is not yet saturated at typical [CO_2_]*i* levels. C3 plants represent ∼85% of plant species globally. Research is also needed to examine the impact of CO_2_-induced stomatal closing during heat stress, given that the CO_2_ response appears to be weaker at high temperatures and heat itself is a signal that promotes stomatal opening (Raschke, 1975).

A better understanding of the molecular mechanisms that enable CO_2_ control of stomatal movements could aid in future molecular enhanced breeding-, engineering-, and/or gene editing-driven improvement of stomatal WUE traits that are better adapted to diverse environments in a high CO_2_ and climate change-challenged world. In recent years, advances have been made toward understanding the molecular mechanisms that cause CO_2_-regulated stomatal movements, with some key questions remaining to be resolved as highlighted here.

**Figure 10:**
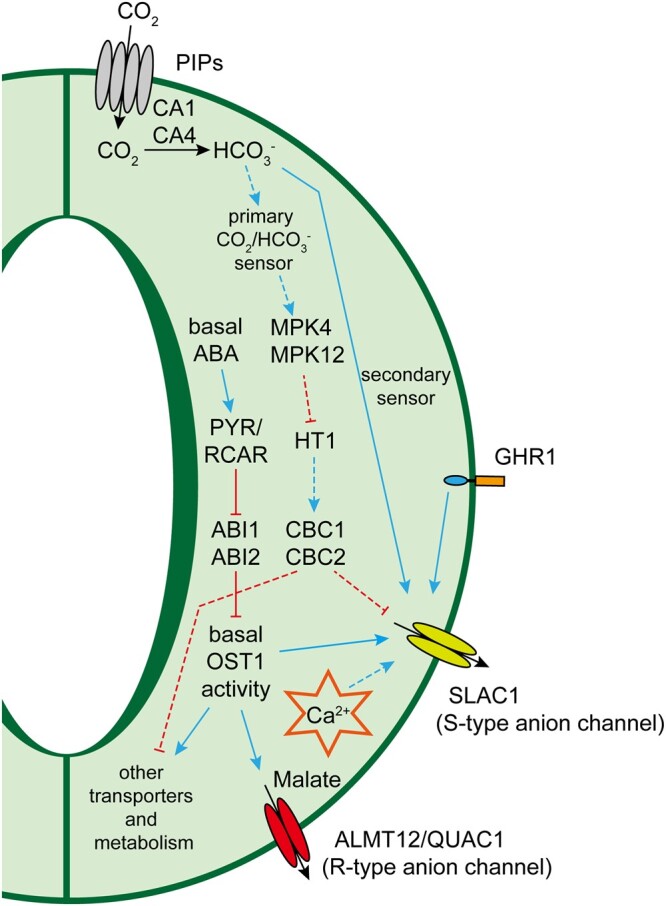
Simplified model for CO_2_ sensing and signaling in stomatal closure. Schematic diagram of signaling components and pathways in CO_2_-induced stomatal closure. Black arrows indicate the directions of transport or enzymatic reaction in response to elevated CO_2_. Blue arrows and red blocks represent positive and negative regulation of high CO_2_-triggered stomatal closure, respectively. PIP2 AQPs (PIPs) and β-carbonic anhydrases (CA1/CA4) could facilitate CO_2_ influx and ${\text{HCO}}_{3}^{-}$ and proton production in guard cells. Protein kinases including MPK4/MPK12, HT1, and CBC1/CBC2 are known early signaling components involved in regulating ion transporters. However, many detailed mechanisms in CO_2_ sensing and signal transduction remain to be investigated as indicated by dash arrows and red dashed blocks. Basal ABA signaling and basal OST1/SnRK2 kinase activities facilitate high CO_2_-induced stomatal closure. A receptor-like pseudokinase GHR1 is required for SLAC1 activation under CO_2_ elevation and several other environmental stimuli.

Studies have shown CO_2_ sensing by guard cells, but also a role for the mesophyll (Mott et al., 2008), in sensing or amplifying the stomatal CO_2_ response. Forward genetic screens have thus far identified guard cell localized molecular mechanisms that function in CO_2_ control of stomatal movements. Thus, the rapid mesophyll-derived signal remains one of the open questions. We discuss guard cell CO_2_ signaling mechanisms and open questions in the following (Figure 10). In brief, CO_2_ entry into guard cells could be facilitated by the CO_2_-permeable PIP2 aquaporins (Mori et al., 2014; for review Zhang et al., 2018a). Carbonic anhydrases in guard cells accelerate the stomatal response to CO_2_ shifts in Arabidopsis, rice, and maize (Hu et al., 2010; Chen et al., 2017; Kolbe et al., 2018). Carbonic anhydrases mediate reversible catalysis of CO_2_ in guard cells to bicarbonate and protons. The βCA4 isoform of carbonic anhydrases is located at the plasma membrane of guard cells and interacts directly with the PIP2;1 aquaporin (for review, Zhang et al., 2018a). Studies have suggested that intracellular bicarbonate (${\text{HCO}}_{3}^{-}$) plays an important role as a second messenger in transducing the CO_2_ signal in guard cells (e.g. Hu et al., 2010). However, the primary bicarbonate/CO_2_ sensor in guard cells that controls stomatal movements has remained elusive (Note that a *secondary* sensor has been identified, as discussed later). Identification of the primary ${\text{HCO}}_{3}^{-}$/CO_2_ sensor that controls stomatal movements will be a key to modifying dynamic CO_2_-dependent WUE.

A forward genetic CO_2_ response screen identified recessive *high temperature 1* (*ht1*) mutant alleles in a RAF-like protein kinase that cause a strong insensitivity to low CO_2_-induced stomatal opening, while showing responsiveness to blue light and ABA (Hashimoto et al., 2006). Mapping of natural variants in WUE and in stomatal regulation have independently identified the mitogen-activated protein kinase MPK12 as a rate-limiting genetic locus (Des Marais et al., 2014; Jakobson et al., 2016). Mechanistic research has shown that in Arabidopsis double mutants of *mpk12* together with the close homolog *mpk4* and in tobacco silencing of the close homolog *NtMPK4* disrupt high CO_2_-induced stomatal closing, while ABA-induced stomatal closing remains intact (Marten et al., 2008; Tõldsepp et al., 2018). Neither MPK12 nor MPK4 protein kinase activities were found to be regulated by CO_2_/${\text{HCO}}_{3}^{-}$ directly in vitro under several examined conditions (Tõldsepp et al., 2018).

Two other RAF-like protein kinases were discovered, CONVERGENCE OF BLUE LIGHT AND CO_2_ (CBC1 and CBC2), for which double mutants show an impairment in low CO_2_-induced stomatal opening (Hiyama et al., 2017). In contrast to recessive *ht1* mutant alleles (Hashimoto et al., 2006), *cbc1/cbc2* double mutants disrupt blue light-induced stomatal opening as well (Hiyama et al., 2017). Therefore, CBC1 and CBC2 are proposed to represent a convergence point of low CO_2_ and blue light-mediated stomatal opening (Hiyama et al., 2017). HT1 can phosphorylate CBC1 in vitro (Hiyama et al., 2017), but the physiological relevance of this reaction for CO_2_-mediated stomatal regulation is unknown.

The carbonic anhydrases (βCAs), HT1, and MPK12/MPK4 proteins function in the early guard cell specific CO_2_ response pathway as positive regulators (βCAs, MPK12/MPK4) and negative regulators (HT1) (Figure 10). Downstream of early CO_2_ signaling, a network of guard cell ion channels, pumps, transporters, and regulators in the plasma membrane and vacuolar membrane as well as metabolic responses (e.g. Flütsch et al., 2020) mediate CO_2_-regulated turgor driven stomatal movements. Elevated CO_2_ activates both slow “S-type” and rapid “R-type” anion channels in guard cells (Raschke et al., 2003). Elevated CO_2_ can be predicted to inhibit plasma membrane proton pumps that drive stomatal opening, although direct evidence is needed. Furthermore, elevated bicarbonate enhances the activity of the S-type anion channel SLAC1 in heterologous cells and in guard cells. Residues in SLAC1 that are required for this response have been identified and the respective SLAC1 mutants show impaired CO_2_ regulation, but intact ABA regulation, of stomatal closing in intact plants, leading to the model that SLAC1 can function as a secondary CO_2_/${\text{HCO}}_{3}^{-}$ sensor in guard cells (Zhang et al., 2018b). Since SLAC1 activation is known to require phosphorylation, the upstream primary CO_2_/${\text{HCO}}_{3}^{-}$ sensor remains, however, unknown (Zhang et al., 2018b).

An important question remains on how early CO_2_ signaling mechanisms control these mediators of stomatal movements. Research suggested that the elevated CO_2_ response is mediated by the ABA receptor signaling pathway (Dittrich et al., 2019). However, CO_2_-regulated stomatal conductance findings in ABA receptor mutants (Dittrich et al., 2019) could not be confirmed using several approaches and showed CO_2_ responsiveness (e.g. Hsu et al., 2018; Zhang et al., 2020). Moreover, recent research has led to the unexpected findings that CO_2_ triggers stomatal closing without further activating SNF1-related protein kinase2 (SnRK2s), including OST1 (Hsu et al., 2018; Zhang et al., 2020), that are activated by ABA. Moreover, basal ABA levels and a basal activity of SnRK2/OST1 protein kinases were found in guard cells, and these are required for amplifying the CO_2_ response even though CO_2_ increases did not rapidly change their activities (Hsu et al., 2018; Zhang et al., 2020). Thus, the link from early CO_2_ signaling mechanisms to downstream targets that mediate stomatal closure remains to be discovered.

There is still much to learn about how CO_2_ regulates stomatal apertures, from CO_2_/${\text{HCO}}_{3}^{-}$ sensors to a biochemical and physiological understanding of the signaling network, which could drive future improvements in WUE of plants including trees, depending on the species, with a need for future quantitative field research (De Kauwe et al., 2021). Furthermore, leaf-level stomatal conductance models are a crucial part of Earth system climate models, and molecular insights could improve the accuracy of these models.

## How can one aquaporin have so many roles in a plant?

### (By Stephen D. Tyerman)

Much research on plant AQPs has assessed their impact on water transport across membranes in response to drought and salinity stress, but low temperature, anoxia, and nutrient stress and combinations also feature (Kapilan et al., 2018). Many studies show that overexpression of an AQP can confer tolerance to these stresses, sometimes multiple stresses, but it is by no means clear how such stress tolerance occurs in the strict context of water transport. Some AQPs, originally designated as true water channels, have been shown to transport multiple substrates besides water, including signaling molecules (e.g. H_2_O_2_), neutral substrates for synthesis (CO_2_, O_2_, and NH_3_), and even ions (Na^+^, K^+^, and NO_3_^−^) (Tyerman et al., 2021) that can all feature in responses to abiotic stress or photosynthesis (Ermakova et al., 2021). AQPs are also under the control of many hormones (Maurel et al., 2021) and are important for regulating growth (Wang et al., 2020a, 2020b). However, genetic evidence indicating AQP involvement in stress resistance, for example, the location of AQP genes under stress-related QTLs, is uncommon (Hostetler et al., 2021a), perhaps due to their complex regulation (Grondin et al., 2016) and multifunctionality.

**Figure 11:**
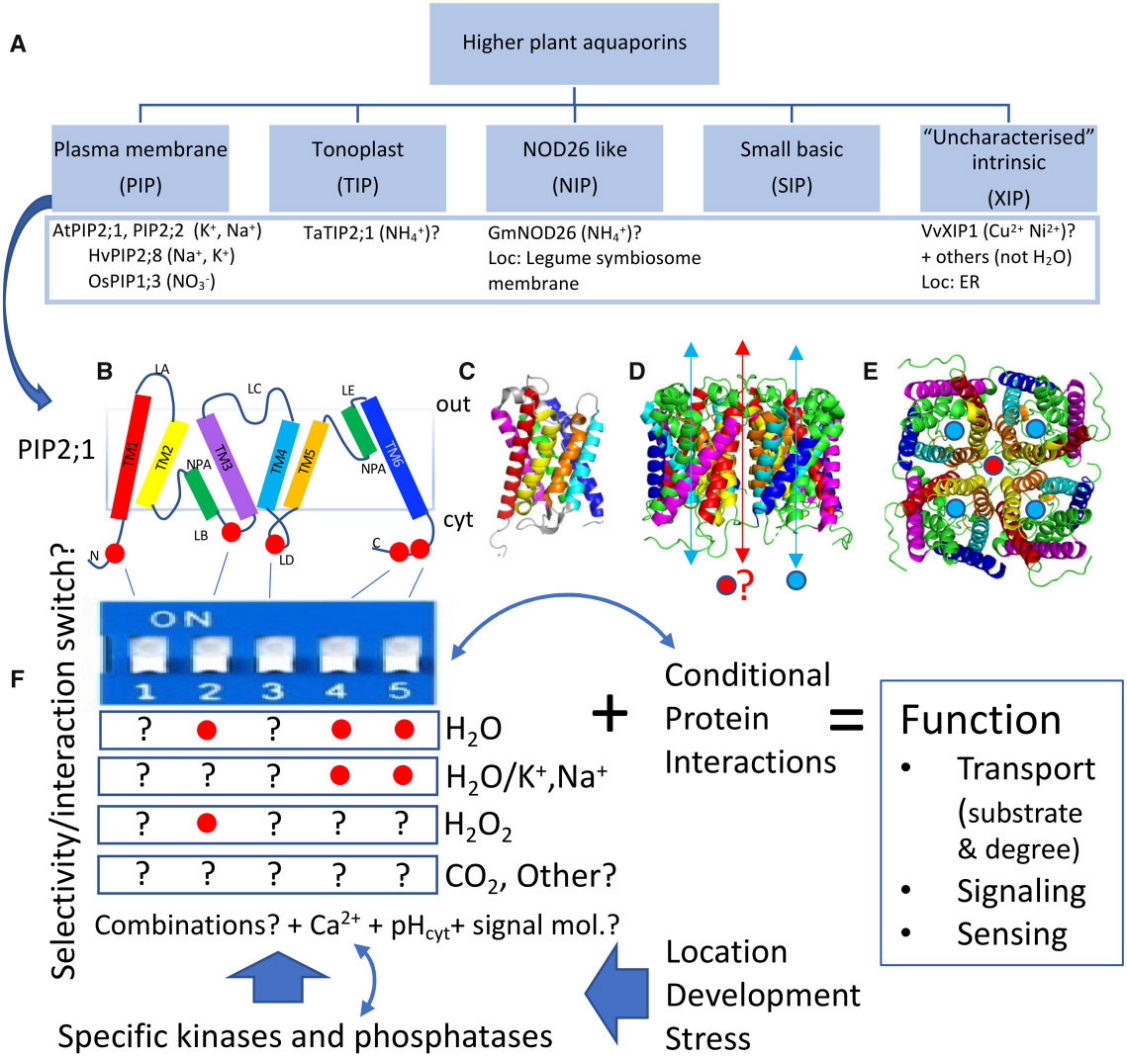
How an aquaporin (AQP) can be multifunctional. A, AQP family with those indicated to transport ions electrogenically (under each subfamily, ? =maybe, Loc= membrane location for specific isoform, note many NIPs are located on the plasma membrane) (Tyerman et al., 2021). B, Features of PIP2;1 showing transmembrane helices (TM1-6), loops (A–E) and half helices forming the central selectivity NPA filter. Red dots = phosphorylation sites. C, Folded monomer structure (SoPIP2;1 PDB 1z98) (Törnroth-Horsefield et al., 2006). D, Homotetramer as the functional unit with four monomeric pores that transport water (blue arrows, dots) and probably H_2_O_2_, also NH_3_ in TIP2;1 (Kirscht et al., 2016) and the central pore (red arrow, dot) implicated in ion transport for mammalian AQP1 (Henderson et al., 2022). AQPs also likely form heterotetramers (e.g. ZmPIP1;2 + ZmPIP2;1) (Chaumont and Tyerman, 2014). E, View normal to the membrane plane on the cytoplasmic face. F, Hypothesis for multifunctionality of PIP2;1. Phosphorylation by specific kinases occurs at one or more of the sites on cytoplasmic loops like a digital dipswitch that determine protein interactions and/or substrate selectivity, for example, ion water reciprocity (Qiu et al., 2020) or H_2_O_2_ permeation (Rodrigues et al., 2017), or protein interactions (Prado et al., 2019). Selectivity is fixed to a certain degree in the monomeric pores by the structure of the ar/R selectivity filter and other residues. The hydrophobic central pore may still allow ion permeation and is proposed to be gated at a low probability dependent on monomer gating (Tyerman et al., 2021). There are likely feedbacks (small blue arrows) due to some substrates and protein interactions also affecting the signaling that determines the kinase/phosphatase activity.

The plant AQPs can be divided into several subfamilies (Figure 11A) and not all of them are good water channels. Within the NIPs (mainly), as well as some PIPs and XIPs, transported substrates include metaloids, protonated organic acids, or metal complexes (Tyerman et al., 2021). Water can also be transported but not always. In each case, the transport is passive (down-hill) in response to the free energy gradient for water, concentration gradients for the solute, or electrochemical gradients for those shown to also pass ions (Tomkins et al., 2021; Tyerman et al., 2021). Multiple substrate transport through the same protein at the same time (e.g. ions and water, CO_2_ and water, and H_2_O_2_ and water) could lead to interactions (Tyerman et al., 2021). It remains to be seen how some substrates permeate and the fifth pore through the center of the tetramer is a candidate for ions and CO_2_ (Figure 11, B–E).

What may still occur, even for passive transport, is rectification (i.e. a greater flow or diffusion in one direction compared with the other depending on the direction and magnitude of the gradient) as is well known for some ion channels. The aromatic/Arginine (ar/R) selectivity filter present on the lumen side of the monomeric pore may give rise to voltage and ion dependence (Mom et al., 2021) that could lead to rectification. Rectification has not been well studied for plant AQPs at the molecular level though it was well studied in the past for water transport across plant cell membranes. This could occur for water to be trapped within the root xylem (Pascut et al., 2021) but reverse flow through a root with reversed gradients would argue against this. Ion flow through AtPIP2;1 and AtPIP2;2 can show rectification with certain divalent cations (Ca^2+^ and Mg^2+^) present (Kourghi et al., 2017).

Some PIP AQPs can account for significant portions of shoot and root hydraulic conductivity (Lpr) (Prado et al., 2019; Ding et al., 2020; Domec et al., 2021). To determine the contribution of AQPs to Lpr requires a combination of sophisticated models and measurements of water transport (Ding et al., 2021; Knipfer et al., 2021). The regulation of root AQPs in response to abiotic stress such as salinity and anoxia via gating or removal from the membrane results in rapid changes in Lpr (Tournaire-Roux et al., 2003; Boursiac et al., 2008). Shoot signals are also implicated in regulation of root AQPs (Chaumont and Tyerman, 2014). Changes in the Lpr can have large effects on stomatal conductance, shoot water relations, and growth (Ding et al., 2020; Knipfer et al., 2021) and perhaps ion content of the shoot related to the reflection coefficient of the root (Knipfer et al., 2021) or the capacity of some AQPs to transport ions. Ultimately, the amount of water relative to the amount of ions transported to the xylem determines the xylem ion concentration for delivery to the shoot. A common signal element that links plant Na^+^/K^+^ ratios under salinity with control of AQPs in the root is the production and transport of H_2_O_2_ (Ma et al., 2011; Martiniere et al., 2019).

Taking the PIP subfamily (with two clades; PIP1 and PIP2) as the best studied example of multifunctionality, one PIP2 isoform can be permeable to water, cations, CO_2_, and the signaling molecule H_2_O_2_. For the cases of cations, water, and H_2_O_2_, phosphorylation on certain residues appears to be key (Figure 11) though there are some variations in the literature regarding the impact on water flow. Arabidopsis AtPIP2;1 has been shown to be required for circadian variation in rosette Lp with 14-3-3 proteins depending on C-term phosphorylation (Prado et al., 2019), for auxin regulation of lateral root outgrowth (Peret et al., 2012), CO_2_ uptake into guard cells with a carbonic anhydrase (Wang et al., 2016), H_2_O_2_ signaling in guard cells dependent on a Loop B phosphorylation (Rodrigues et al., 2017), and univalent cation transport dependent on C-term phosphorylation (Qiu et al., 2020). The very similar AtPIP2;2, also permeable to cations but with increased sensitivity to Ca^2+^ compared with AtPIP2;1 (Kourghi et al., 2017), contributes to Lpr derived from root exudation (Lp^rex^) but not Lpr derived from pressure gradients (Lpr^pres^) (Javot et al., 2003). Root exudation is important for xylem water continuity and may be more complicated than ion pumping into the xylem with the subsequent osmotic gradient driving flow (Schenk et al., 2021). Interestingly, AtPIP1;2 when knocked out reduces Lpr^pres^ but not Lp^rex^ pointing to an interesting division of labor that may depend on different locations of the two AQPs (Postaire et al., 2010). Another PIP2 from rose is implicated in a drought signaling hub releasing a membrane bound transcription factor depending on phosphorylation status and environmental signals to control growth under water stress (Zhang et al., 2019). The well-studied maize PIP2;5 is also multifunctional being implicated in guard cell signaling for ABA responses due to H_2_O_2_ permeation (Ding et al., 2021) but also for its water permeation in the root (Ding et al., 2020).

Many introductions to papers on AQPs describe them as exclusively important for water transport, implying that there is only water transport. But the situation now emerging is far more complex and the PIPs seem to be implicated in various aspects of plant water relations that are more than just water transport across a membrane. This makes interpretation of phenotypes in both reverse and forward genetics rather challenging. The question as to how a single AQP can function in such a broad range of cell types with different transported substrates or signaling roles is addressed by the hypothesis in Figure 11F. The answer may lie in a digital-like switch through posttranslational modifications and signaling pathways combined with many and varied protein interactions.

## How do plants balance growth and abiotic stress responses?

### (By Taishi Umezawa)

**Figure 12:**
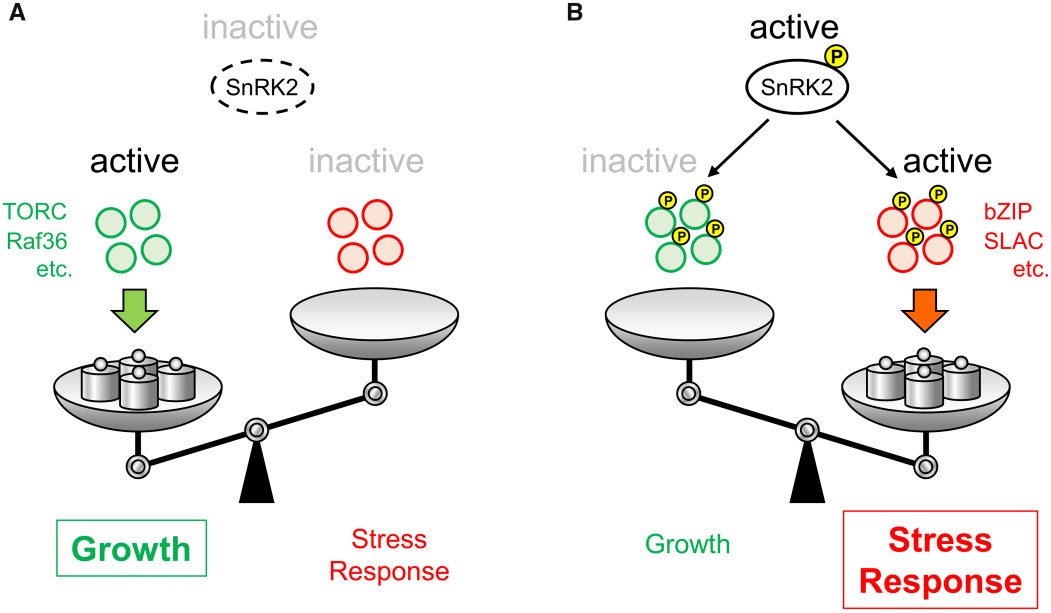
SnRK2 kinases regulate growth and stress response under drought. A, Under normal conditions, plants use energy to grow, and TORC or Raf36 are involved in this process. B, Under drought conditions, SnRK2s are activated and phosphorylate substrates, for example, bZIP transcription factors or slow anion channels (SLAC), to induce stress responses. SnRK2s also phosphorylates TORC or Raf36 to inhibit plant growth.

Plant growth is affected by many environmental factors that alter the balance of energy use (Crepin and Rolland, 2019). Under favorable environmental conditions, plants are able to assign the energy obtained from photosynthesis to growth, especially during the vegetative growth phase. Conversely, under adverse environmental conditions, plants must redirect their energy to stress responses to overcome the challenge and ensure individual survival. However, when stress conditions are prolonged, it is not always a suitable strategy to inhibit growth indefinitely. Under such conditions, it may be advantageous for plants to maintain growth to some extent, or to switch to their reproductive stage to preserve the next generation. It is likely that plants have evolved to adjust their energy balance precisely in response to abiotic stresses, because they are often forced to make such decisions throughout their life cycles. The question is how plants tilt the balance toward stress responses or toward growth regulation (Figure 12).

In the case of drought, plant responses to this stress have been divided into three alternative strategies: drought tolerance, drought avoidance, and drought escape (Kooyers, 2015). For short-term drought, drought avoidance or tolerance can be effective. For example, it is well known that plants can quickly close their stomata to prevent water loss from the leaf surface. Similarly, plant cells can rapidly adjust their osmotic potential to maintain water status (Zhu, 2002; Yamaguchi-Shinozaki and Shinozaki, 2006). Molecular mechanisms that turn on such stress responses have been well studied, especially responses induced by the phytohormone ABA. The major ABA signaling pathway consists of three core components: ABA receptors (PYL/RCAR), Clade A of the type 2C protein phosphatases (PP2C), and SnRK2 kinases (Cutler et al., 2010; Umezawa et al., 2010). Under normal conditions, PP2Cs directly dephosphorylate and inactivate SnRK2s (Umezawa et al., 2009; Vlad et al., 2009). In response to ABA, this inhibition is abated and active SnRK2s can phosphorylate various protein substrates to induce ABA-associated responses including stomatal closure and large-scale changes in gene expression. Since SnRK2s are central players in drought responses, many studies have used SnRK2s as a starting point to identify signaling proteins involved in ABA or osmotic stress signaling (Wang et al., 2018a; Kamiyama et al., 2021).

In addition to their central role in ABA signaling, SnRK2s also function to regulate plant growth under drought stress conditions. Recently, SnRK2s were shown to directly phosphorylate Raptor, a component of the TOR complex (TORC) that regulates plant growth (Figure 12). Under stress conditions, phosphorylation of Raptor by SnRK2 resulted in dissociation of TORC and inhibition of plant growth (Wang et al., 2018a, 2018b). Separately, a recent study demonstrated that Raf36, a group C Raf-like protein kinase, promotes growth under normal conditions, and is degraded in response to ABA by SnRK2-dependent phosphorylation (Kamiyama et al., 2021). These two examples highlight an important role for SnRK2s not only for ABA-dependent stress responses, but also for mediating growth inhibition under short-term and severe drought stress. In addition to the SnRK2 pathway, Clade E Growth-Regulating (EGR) phosphatases and Microtubule-Associated Stress Protein 1 are involved in growth regulation during drought stress (Longkumer et al., 2022). It is expected that identifying and functionally characterizing additional SnRK2 or EGR substrates will aid our understanding of the mechanisms of growth inhibition under drought stress.

In nature, sudden and severe drought stress on plants is not likely to occur. In most cases, drought stress gradually increases in stages. When drought stress is mild and prolonged, it is likely not beneficial for plants to spend energy only on stress responses, and under such conditions, plants may continue to grow as part of their drought avoidance or escape strategies (Kooyers, 2015). For example, root growth often increases during mild drought as a means to increase access to available water. In rice, a root angle QTL, DRO1, was shown to be effective for selection of drought tolerance in rice, demonstrating that drought avoidance is one of the promising breeding targets for drought resistance (Uga et al., 2013).

In the drought escape response, plants accelerate floral development and the transition to the next generation (Kooyers, 2015). ABA is involved in early flowering as a drought escape response, and multiple pathways are proposed to link ABA and flowering (Martignago et al., 2020). For instance, several bZIP transcription factors, AREB/ABFs, are phosphorylated by SnRK2s and involved in drought escape (Hwang et al., 2019). In addition, previous studies proposed that the photoperiodic flowering pathway, consisting of GIGANTEA (GI), CONSTANS, and FT, is essential for early flowering (Riboni et al., 2013, 2016). However, the connection between ABA and GI-dependent FT pathway is still under investigation.

Drought stress is not constant and stress intensity fluctuates over time in nature. Once drought stress reaches a certain level, plants cross a threshold and change the balance between growth and stress response. It will be important to identify the molecular switch involved in such a stage-gate of drought stress. Furthermore, if the intensity of drought stress changes frequently, plants can acquire stress memory. It is known that some epigenetic modifications could be involved in stress memory (Sharma et al., 2022) and may regulate intra- or inter-generational responses to stress conditions. This is another topic to be clarified.

As discussed in this section, plant growth regulation is complex, and current knowledge of plant growth regulation under stress conditions is just beginning to scratch the surface. Further studies will be required to understand how plants balance stress response and growth regulation, and in depth understanding of such mechanisms could facilitate molecular breeding for yield and quality of agricultural production under drought conditions.

## Proline metabolism: protector, scavenger, or executioner?

### (By Paul E. Verslues)

**Figure 13:**
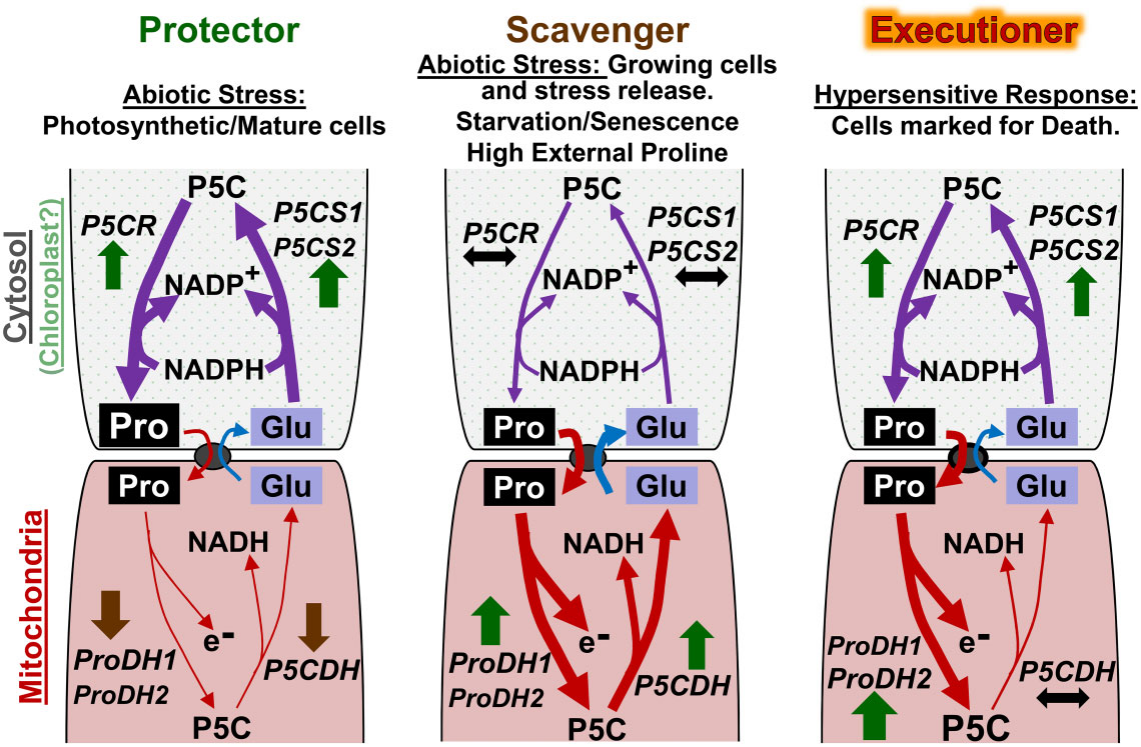
The protector, scavenger and executioner modes of proline metabolism and the conditions or tissue in which they are observed. Up and down arrows (red, brown, or gray) indicate changes in gene expression compared with unstressed control. In most cases where data are available, protein levels of the corresponding enzyme change in the same direction. Thickness of the red or purple arrows represents predicted relative metabolic flux through different steps in proline metabolism. Abiotic stress refers to drought, freezing or high salt treatments where sustained accumulation of free proline is observed in many plant species.

Proline is highly soluble and zwitterionic, hallmarks of compatible solutes that accumulate to reduce cellular osmotic potential while also protecting protein and membrane structure from dehydration-induced damage. However, making proline is not the only impact of proline metabolism and the protective role of proline itself is not the only purpose of stress-induced proline accumulation (Bhaskara et al., 2015; Alvarez et al., 2022). The proline cycle (Figure 13) consists of synthesis from glutamate by Δ^1^-pyrroline-5-carboxylate (P5C) synthetase (P5CS) and P5C reductase (P5CR) while proline catabolism back to glutamate is catalyzed by proline dehydrogenase (ProDH) and P5C dehydrogenase (P5CDH). In Arabidopsis, *P5CS1* and *ProDH1* are the most stress-responsive of the proline cycle genes and the proteins they encode catalyze the rate-limiting steps of proline synthesis and catabolism, respectively. One protective function of the proline cycle is to regenerate NADP^+^ to help ensure the supply of a safe electron acceptors during stress and when leaf CO_2_ becomes limited (Hebbelmann et al., 2011; Sharma et al., 2011; Signorelli, 2016). Both P5CS1 and P5CR prefer NADPH over NADH as an electron donor (Giberti et al., 2014; Forlani et al., 2015; Sabbioni et al., 2021) and *p5cs1* mutants have a reduced NADP^+^/NADPH ratio during low water potential (*ψ*_w_) stress (Sharma et al., 2011). *p5cs1–4* also has substantial changes in photosynthesis-related gene expression (Shinde et al., 2016). How such a proline synthesis-photosynthesis redox link could work depends on the subcellular localization of P5CS1 and P5CR and how it is affected by stress. P5CS1 is likely to be localized in the cytoplasm; however, this is ambiguous as both cytoplasmic (Funck et al., 2020) and chloroplast, or chloroplast-associated, localization (Székely et al., 2008) have been reported. Similarly, fluorescently tagged P5CR was localized mainly in the cytoplasm (Funck et al., 2012) while biochemical or immunoblot assays indicated that it could also be present in plastids (Szoke et al., 1992; Murahama et al., 2001). The preference of P5CS1 and P5CR for NADPH is also consistent with the proposal that proline synthesis is linked to the pentose-phosphate pathway in a redox cycle (Hare and Cress, 1997). However, this proposal has been little tested.

ProDH1 and P5CDH are also scavengers in that plants can use proline as an alternative respiratory substrate during senescence and dark-induced starvation (Zhang and Becker, 2015). This is facilitated by the fact that ProDH transfers reductant directly to ubiquinone via its FADH cofactor (Zheng et al., 2021). During stress recovery (after restoration of water supply), when proline levels rapidly decline, or in response to exogenous proline, *ProDH1* and *P5CDH* expression is induced (Figure 13) and proline catabolism can feed so much reductant into mitochondrial electron transport that some of it needs to be vented off by alternative oxidase to prevent oxidative stress (Oh et al., 2022). The rapid catabolism of proline after re-watering may be a way to channel the nitrogen from proline to other amino acids needed during the resumption of growth.

Interestingly, *p5cs1* mutants, which have greatly reduced proline accumulation, and *prodh1* mutants, which have increased proline accumulation, have similar low *ψ*_w_-sensitive phenotypes (Sharma et al., 2011; Bhaskara et al., 2015). Low *ψ*_w_ stress leads to *ProDH1* downregulation in most of the plant tissues. However, meristematic and growing cells have steady or increased *ProDH1* and *P5CDH* expression during low *ψ*_w_ stress (Sharma et al., 2011). This indicates that the proline cycle, with the synthesis versus catabolism sides of the cycle spatially separated, can also facilitate the movement of reducing potential, stored in the form of proline, away from photosynthetic tissue where it is in excess, to root and meristem tissue where proline is used for energy metabolism or osmotic adjustment (or in other words: to be a good protector, it is important to know when to also be a scavenger).

Proline metabolism shows its executioner side during the hypersensitive response (HR) to incompatible pathogen infection (Figure 13). Proline accumulation mediated by P5CS2 and proline catabolism by ProDH1 and ProDH2 is required for HR-induced cell death and associated ROS burst (Fabro et al., 2004, 2016; Cecchini et al., 2011; Senthil-Kumar and Mysore, 2012). During infection, cells marked for death have up-regulated expression of *P5CS2*, *P5CR*, and *ProDH1* but *P5CDH*, leading to partial proline catabolism that is associated with cell death (Alvarez et al., 2022; Figure 13). Whether the cell death is caused by proline-dependent ROS production or a yet unknown signaling function of P5C (or a combination of the two) is unclear. P5C is the common intermediate of both proline synthesis and catabolism and it has also been proposed that P5C may be exported from the mitochondria and used for proline synthesis in the cytoplasm, thus forming a proline–P5C cycle which could amplify ProDH-dependent ROS production in the mitochondria (Miller et al., 2009). However, evidence supporting such a P5C cycle in plants is circumstantial and a mitochondrial P5C translocator has not been identified.

Whether proline metabolism operates in protector, scavenger, or executioner mode depends on unknown environmental and metabolic signals. Thus, proline metabolism is not only interesting in its own right in terms of how it protects or kills plant cells, it is also a useful model to discover new aspects of stress signaling. For example, what sensing and signaling events occur during drought stress to allow high levels of proline to accumulate without inducing *ProDH1* and without having proline metabolism switch into executioner mode to promote cell death (Miller et al., 2005)? This is especially interesting as the sensing and upstream signaling mechanism(s) plants use to detect and respond to reduced water availability during drought stress remain unknown. For proline metabolism, the relative fluxes through different parts of the proline cycle (indicated by different arrow thicknesses in Figure 13) are inferred from gene expression or protein levels of proline metabolism enzymes but there is little information on actual metabolic flux rates through the proline cycle under different conditions. This is important information for determining the conditions where proline catabolism is rapid enough to either significantly contribute to respiration (scavenger mode) or significantly increase ROS levels (executioner mode) and how this is coordinated with mitochondrial mechanisms to dissipate excess reducing potential, including alternative oxidases and uncoupling proteins. Posttranslational modifications of P5CS1 and ProDH1 (Alvarez et al., 2022) or interactions with regulatory proteins (Ren et al., 2018) are likely to affect their enzymatic properties but the roles of such factors in controlling the protector–scavenger–executioner modes of proline metabolism are unknown. For P5CS1 and P5CR, knowledge of their subcellular localization is also strikingly limited. Surprisingly, Savoure and co-workers have reported that a *prodh1prodh2* double mutant, in which the only two ProDH genes in the Arabidopsis genome are knocked out, is viable despite having no known way to catabolize proline (Alvarez et al., 2022). Is there a metabolic work around that allows these plants to metabolize proline? And, Figure 13 depicts a mitochondrial proline–glutamate exchanger and such an activity, along with that of mitochondrial proline importer(s), has been biochemically observed (Di Martino et al., 2006); however, the genes encoding these activities remain unknown.

Perhaps one of the most striking paradoxes of proline and stress, given all the evidence of the importance of proline to stress resistance, is that some plants apparently do without it. For example, most Arabidopsis accessions accumulate high levels of proline during low *ψ*_w_ stress; but a few have very low levels of P5CS1 and greatly reduced levels of proline accumulation similar to *p5cs1* knockout mutants (Kesari et al., 2012). Are these accessions more sensitive to drought (or salt) stress, or have they found a substitute for the stress-protective (and executioner) effects of proline metabolism? Also, some plants adapted to chronically dry conditions have relatively low levels of free proline accumulation but instead convert proline to proline betaine or hydroxy-proline betaine as these may be more effective osmoprotectants (Hanson et al., 1994). However, the implications of this conversion for the proline cycle are unknown and these compounds are likely to be more difficult to catabolize, thus impeding redeployment of nitrogen and reducing potential when the stress subsides.

Is proline metabolism a protector, scavenger, or executioner? It depends. Depends on what is the real question; a question whose answer will reveal much about the sensing, signaling, and metabolic mechanisms that plants use to cope with abiotic stresses that are of increasing concern for a warming and changing world.

## Temperature sensing: how do plants adapt to different climates?

### (By Philip A. Wigge)

A remarkable feature of plants is their ability to adapt to a wide range of climates, occupying almost every niche from the tropics and hot springs to polar regions. To do this, plants have evolved an array of responses to temperature, over multiple scales, from minutes to months, which enable a suite of developmental and cell biological responses to maximize survival. Understanding how plants are able to adapt to different climates is a major open question, and of particular relevance during a period of unprecedented rapid global heating (Battisti and Naylor, 2009).

Broadly, we can consider active and passive responses to temperature. Passive responses refer to adaptations such as membrane fluidity and protein stability. Proteins at high temperature tend to denature and unfold. Thermophiles therefore have proteins with increased ionic interactions and a larger stable hydrophobic core. At low temperature, there is reduced molecular motion due to low entropy and enthalpy, and psychrophilic organisms adapt by having proteins with fewer salt bridges and hydrogen bonds to facilitate flexibility (Brininger et al., 2018).

A major strategy of plants has been to evolve active temperature sensing and response pathways. These enable the anticipation of both seasonal temperature shifts as well as shorter term temperature stresses. Temperature measurements over the year, in concert with photoperiod, enable plants to avoid unfavorable seasons in a dormant stage, and grow and flower during suitable months. While there is enormous diversity in the habitats and climates that plants have adapted to, conservation of major signaling components appears to be a common theme.

Broadly, two major approaches have been followed to identify the genes and mechanisms underlying adaptation to different temperatures. From a population perspective, it is possible to harness the power of genetics to identify genomic regions and loci that show signatures of natural selection in populations from different locations (Hancock et al., 2011). Another strategy is to identify the underlying temperature sensors in genetic screens or using a candidate gene approach. This method is complicated by the often pleiotropic and redundant nature of temperature signaling pathways, reflecting their central role in many essential responses. The use of carefully designed and controlled temperature screens can, however, overcome some of these issues. Sensors may then be investigated for natural variation in the context of their mode of action. This approach has the advantage that it is targeted and enables a test of functionality, as well as identification of variation which directly impacts temperature signaling. We will discuss some well-studied temperature responsive networks that suggest pathways to adaptation.

### Vernalization

**Figure 14:**
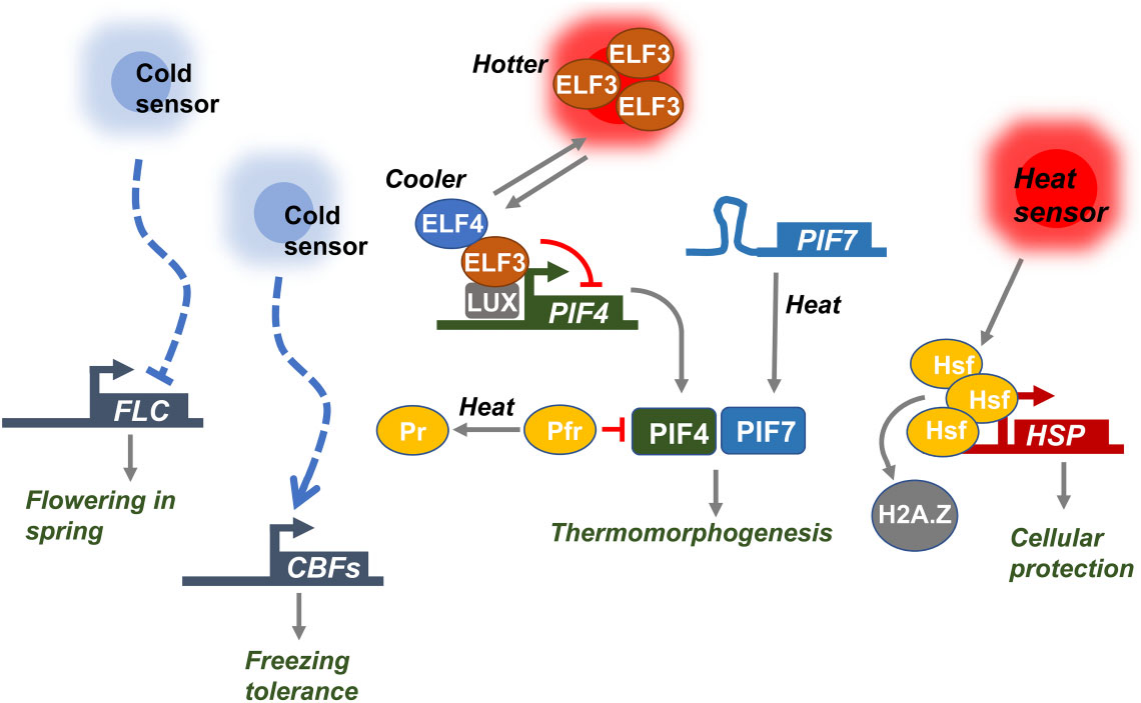
Temperature response mechanisms. Temperature controls many key adaptive traits in plants such as freezing tolerance, growth, flowering, and heat stress protection. Vernalization results in the stable repression of expression of the floral repressor *FLC*. Thermomorphogenesis integrates temperature information from at least three distinct biophysical responses: protein phase separation of ELF3, thermal reversion of phyB (Pfr to Pr) and enhanced translation of *PIF7* in warm conditions. Heat stress also induces the induction of protective heat shock proteins via the activation of HSFs via an unknown mechanism.

Vernalization, the response of plants to prolonged cold, is a classical temperature response in Arabidopsis. Extensive natural variation exists at the level of the major regulators *FLC* and *FRI* (Shindo et al., 2005; Figure 14). The complex nature of *FLC* silencing in response to cold via a proposed antisense RNA also lends itself to natural variation and modulation in terms of the extent and duration of cold requirement (Duncan et al., 2015).

### Thermomorphogenesis

Accelerated growth in response to warm temperature and flowering in Arabidopsis is termed thermomorphogenesis. This process is dependent on enhanced activity of PIF4 (Quint et al., 2016). PIF4 is regulated posttranslationally by the thermosensor phytochrome B (phyB). phyB measures temperature through its dark reversion rate (Jung et al., 2016a; Legris et al., 2016). Different rates of dark reversion can evolve orthogonally to light sensing and cause a corresponding change in temperature sensitivity. This could enable thermorphogenesis to be tuned to the local environment. In addition to the identification of phyB in a natural variation screen for thermal responsiveness, the gene *EARLY FLOWERING3* (*ELF3*) was also identified (Box et al., 2014; Figure 14). ELF3 contains a thermoresponsive predicted prion domain (Jung et al., 2020). This has been shown to have extensive natural variation in the length of a polyQ repeat (Undurraga et al., 2012), and variation both within Arabidopsis as well as with other plants such as *Brachypodium distachyon* directly changes temperature responsive behavior. The polyQ region is encoded by a short tandem repeat (STR), which can expand or contract during replication through DNA polymerase slippage, enabling the generation of functional variation in ELF3 (Kashi and King, 2006). Since the thermal responsiveness of the protein is proportional to the size of the repeat, this may allow for adaptation to different temperature ranges. It will be interesting to see if this STR-based mechanism occurs in other temperature-responsive proteins, as has been suggested for Drosophila (Sawyer et al., 1997). An additional thermosensory mechanism is displayed by the RNA secondary structure in the 5′ UTR of *PIF7*, which facilitates enhanced translation at higher temperatures (Chung et al., 2020). Since 5′ UTR sequences can evolve independently of protein function, this represents a mechanism to alter the levels of protein rapidly in response to temperature. It is not known if this is a widespread mechanism in plants.

### Temperature stress

Survival of freezing stress is mediated by the CBF transcription factors in Arabidopsis, which are activated by cold perception (Jaglo-Ottosen et al., 1998). Heat stress responses are activated by the conserved heat shock factors (HSFs), which activate protective heat shock proteins. In plants, HSFs have undergone a remarkable expansion from 1 to 3 family members in yeast and mammals to 21 members in Arabidopsis (Baniwal et al., 2004). The basis for this is not clear, but suggests an important role for this family of TFs in mediating plant survival (Figure 14).

Critical to understanding adaptation to climate will be determining how many temperature sensors are present in plants. The very distinct genetic and physiological responses to cold during vernalization and cold stress suggest independent sensors, while the heat stress is similarly independent from thermomorphogenesis. Nevertheless, it is plausible to propose as few as 10–20 distinct temperature sensors may account for most of the transcriptional responses to warm and cold temperatures observed in Arabidopsis. Thermomorphogenesis is perhaps the most well-studied system, and in this case, it is interesting that multiple discrete sensors act at different scales and levels (transcriptional, translation, and posttranslation). Such redundancy may represent a mechanism to filter the inherent noise from temperature signals. How temperature information is remembered and integrated over many months is also not understood. Heat stress in the field occurs in a complex environmental context, often with drought stress, and therefore how these various stresses are integrated is important. Analysis of natural variation and field studies of a broader range of plants will also be critical for understanding mechanisms by which sequence variation can achieve rapid changes in the temperature response range of thermosensors. This knowledge will enable the rational editing of crops to enhance thermal resilience.

## Notes

Authors are listed alphabetically (with exception of the coordinating editor), article sections are arranged alphabetically based on first author of each section. All authors contributed to writing the article.

The author responsible for distribution of materials integral to the findings presented in this article in accordance with the policy described in the Instructions for Authors (https://academic.oup.com/plcell) is: Paul E. Verslues (paulv@gate.sinica.edu.tw).
